# Blunted diurnal cortisol slope as a risk marker for 9-year executive function decline: amplification by daily stress and worry severity

**DOI:** 10.1093/geronb/gbag178

**Published:** 2026-08-31

**Authors:** Nur Hani Zainal, Stephanie Noble

**Affiliations:** Department of Psychology, National University of Singapore, Kent Ridge Campus, Singapore; Yeo Boon Khim Mind Science Centre, National University of Singapore, Kent Ridge Campus, Singapore; Department of Psychology, Northeastern University, Boston, Massachusetts, United States; Department of Bioengineering, Northeastern University, Boston, Massachusetts, United States; (Psychological Sciences Section)

**Keywords:** Distal risk factor, Generalized anxiety disorder, Hypothalamic–pituitary adrenal axis, Longitudinal

## Abstract

**Objectives:**

Cortisol dysregulation, reflective of hypothalamic–pituitary–adrenal (HPA) axis dysregulation, has been linked to executive functioning (EF) decline. However, substantial effect-size heterogeneity across studies highlights the potential role of psychosocial moderators. We investigated whether the severity of perceived daily stressors and chronic worry moderated the relationships between baseline cortisol dysregulation and 9-year EF.

**Methods:**

Community adults (*N *= 1,617) completed 4-day, 4-times-daily salivary cortisol sampling and an 8-day interview-driven daily diary at baseline. Three cortisol metrics were computed: area under the curve (AUC), cortisol awakening response (CAR), and diurnal cortisol slope (DCS). Linear mixed models determined whether daily stressor or chronic worry severity moderated associations between baseline cortisol metrics and 9-year EF, adjusting for baseline EF.

**Results:**

Daily stressor severity significantly moderated the relationship between DCS–but not AUC or CAR–and 9-year EF (Cohen’s *d *= 0.113). Flatter DCS was associated with poorer EF across all stressor severity levels, although the observed numerical attenuation at higher severity was not statistically confirmed. Chronic worry severity was significantly associated with lower 9-year EF across all cortisol metrics and significantly moderated the CAR-to-EF relationship (*d* = −0.176). Exploratory analyses suggested that a flatter DCS was associated with worse EF at higher worry severity, but this interaction was not significant.

**Discussion:**

These outcomes broadly align with moderation, not mediation, suggesting that psychosocial stress and worry can modulate, rather than yield, cortisol-linked relationships with EF. Pending replication, HPA-axis circadian modulation and chronic worry severity can indicate viable targets for prevention research.

Executive functioning (EF) is a prominent aspect of cognitive aging and one of the most impactful sources of role impairments in midlife and older adulthood. EF encapsulates a set of higher-order cognitive control processes that govern goal-directed behaviors. It includes inhibition (overriding autopilot responses), set-shifting (flexibly transitioning between cognitive task sets), and updating (real-time maintenance and adaptation of working memory [WM]; Miyake et al., 2000). These basic abilities are essential for daily functioning in older adults: they direct financial management, decision-making, emotion regulation, health behaviors, and medication adherence. As the median age of societies is predicted to increase from 26.6 years in 2000 to 37.3 years in 2050 and 45.6 years by 2100, the societal burden of EF decline grows, signaling a key public health challenge (Khan et al., 2024). The person-level lifetime burden of EF deterioration has been estimated at $124,000 per capita, totaling $627 billion in the United States alone (Gracner et al., 2025), underscoring the significance of determining the biopsychosocial risks of EF decline. Critically, about 45% of dementia cases worldwide worldwide have been linked to modifiable risk factors (e.g., physical inactivity, hypertension, depression; Livingston et al., 2024), highlighting plausible avenues for risk-reduction strategies.

Cortisol dysregulation, reflecting disturbances in the hypothalamic–pituitary–adrenal (HPA) axis, has been theorized to be a long-term correlate of EF decline. Three established cortisol metrics indicate unique aspects of HPA axis functioning (Adam et al., 2017): total area under the curve (AUC) models global waking cortisol production; cortisol awakening response (CAR) indicates the acute increase in cortisol within 30–45 min after awakening, signaling HPA reactivity; and diurnal cortisol slopes (DCS) indicates the cortisol decline rate from morning to before bedtime, where a flatter slope marks compromised circadian HPA regulation. Prolonged HPA disturbances have been posited to neurotoxically impact the prefrontal cortex (PFC) and hippocampus through modified glucocorticoid receptor communication. These processes have been implicated in glucocorticoid-linked negative feedback modulation, glutamatergic communication, and neuroplasticity in EF-relevant neural pathways (Popoli et al., 2011). In addition, these pathways have been linked to glucocorticoid receptor-related epigenetic modifications over extended periods (Lightman et al., 2020; Lupien et al., 1998). Together, these paths suggest that cortisol dysregulation, particularly flatter DCS, may be linked to long-term EF consequences, though the strength of these associations may vary across participants and psychosocial settings.

Prospective evidence supports this hypothesis, but findings vary. An individual-participant data meta-analysis of five cohorts found that higher bedtime cortisol was associated with poorer fluid cognitive capacity (Gardner et al., 2019). It also found that flatter DCS was associated with worse fluid reasoning. Among older adults, higher AUC levels were longitudinally associated with poorer delayed recall and verbal learning after 5 years (Segerstrom et al., 2016). Other cohort evidence demonstrated that blunted DCS was associated with reductions in verbal fluency and global cognition over 5–9 years (Tsui et al., 2020). These patterns persisted even after accounting for baseline EF scores. Although these patterns align with the potential that cortisol dysregulation precedes neurocognitive alterations, alternative accounts (e.g., bidirectionality, pre-existing cognitive deterioration, and common third-variable risk factors) still remain in observational designs. However, effect sizes differed greatly across studies (Amendola et al., 2025; Gardner et al., 2019). Such heterogeneity in effect sizes suggests that individual and contextual moderating variables may affect the strength of the cortisol-to-EF relationship. Therefore, determining moderating variables can clarify for whom and under what psychosocial contexts cortisol dysregulation has the strongest relationship with long-term EF outcomes.

Perceived daily stressor and chronic worry severity are theoretically based variables to moderate the prospective link between cortisol dysregulation and long-term EF outcomes. Of the dimensions whereby daily stress research has defined stress encounters (e.g., appraised stressfulness, emotional reactivity, and stressor exposure; Almeida et al., 2009), the current study attends to perceived stressor severity. This is defined as the self-rated stressfulness of encounters happening on a specific diary day, aggregated across simultaneous stressor categories. The theories posited here differentiate the functions of these variables: cortisol dysregulation, measured at baseline, serves as a biological correlate of HPA-axis functioning, thought to be linked with long-term EF outcomes. Daily stressor severity, defined as the appraised stressfulness of simultaneous everyday events, and chronic worry severity function as psychosocial moderators. These moderators are hypothesized to interact with pre-existing cortisol dysregulation in their relationships with EF-linked neurobiological pathways, rather than independently implicating HPA dysregulation. Such positions are contextualized inside the biological sensitivity model: persons with flatter DCS can have a compromised ability to recover to homeostasis after somatic hyperactivation. High daily stressor severity and chronic worry can impede the return to homeostatic modulation, potentially amplifying the neural outcomes associated with cortisol dysregulation over time. Based on the allostatic load framework (McEwen, 2017), cortisol dysregulation and psychosocial stress have each been thought to add to allostatic overload. Nonetheless, their combined impacts may generate neurobiological outcomes that supersede the sum of their individual effects (Buller-Peralta et al., 2024). This two-factor interaction viewpoint posits moderation rather than mediation: daily stressors and chronic worry severity are expected to magnify the relationship between pre-existing cortisol dysregulation and EF outcomes, rather than yield or explain that dysregulation. Although a mediation path is also theoretically viable (e.g., stress → next-day DCS → future EF), it was not the focus of the current study because the retrieved data contained cortisol only at baseline. Neurobiologically, high daily stressor exposure and the severity of chronic worry may adversely affect recovery-based pathways, such as glucocorticoid receptor resensitization, hippocampal neurogenesis, and PFC-linked top-down inhibition of HPA hyperreactivity (Sheth et al., 2017). These paths are posited to protect against the cumulative neural effects of heightened cortisol exposure over time (Myers et al., 2014). Collectively, high daily stressors and chronic worry severity are posited to lower the physiological threshold at which cortisol dysregulation is linked with noteworthy long-term EF deficits.

Initial empirical data align with this moderation framework, but no prior research has directly evaluated this conjecture in a longitudinal design. Cross-sectional data suggested that cortisol dysregulation was related to cognitive dysfunction, particularly in older adults with, but not without, generalized anxiety disorder (GAD) symptoms (Rosnick et al., 2013). Rickenbach et al. (2014) found that daily stress amplified the relationship between cognitive decline across a decade and everyday memory failures, offering initial support for the idea that psychosocial stress moderates relationships between cognitive vulnerability and daily cognition. Moreover, experimental work has shown that anxiety moderated the negative impacts of acute stress on WM under higher cortisol levels (Hood et al., 2015). However, cross-sectional designs hinder causal temporal inference, whereas experiments provide information across timescales of seconds or minutes rather than months or years (Shields et al., 2016). Furthermore, previous studies have modeled these relations as linear, although, based on theory and empirical research, nonlinear boundary conditions in HPA physiology are plausible (Frost et al., 2023). This study addresses these gaps by evaluating whether baseline perceived daily stress or the severity of chronic worry nonlinearly moderates the prospective relationship between cortisol dysregulation and residualized 9-year EF.

Given the theoretical models and empirical data outlined above, we evaluated two hypotheses. We hypothesized that greater cortisol AUC, more blunted CAR, and flatter DCS would each significantly interact with greater daily stressor severity to predict lower residualized 9-year EF scores (Hypothesis 1). Moreover, we hypothesized that higher cortisol AUC, more blunted CAR, and flatter DCS would each significantly interact with greater baseline chronic worry severity to forecast lower residualized 9-year EF scores (Hypothesis 2). Based on theory and research, we anticipated that the interaction comprising DCS would produce the largest effect sizes, since this metric particularly captures the circadian modulatory dimension of HPA axis functioning, which is intimately linked to long-term EF-implicated neural pathways.

## Method

### Participants

Participants were community-dwelling, noninstitutionalized adults who were enrolled in the Midlife Development in the United States (MIDUS) study (Ryff et al., 2019a). At baseline, 4,963 participants were enrolled in the MIDUS 2 (M2; 2004–2009; Radler & Ryff, 2010), and 3,294 participated in the MIDUS 3 (M3; 2013–2014) wave approximately 9 years later (Ryff et al., 2019a). Of the baseline M2 individuals, 1,617 participants completed all three substudies, including the Biomarker Project (Ryff et al., 2019b), the Cognitive Project (Ryff & Lachman, 2017), and the Daily Stress Project (Ryff & Almeida, 2017), at both time points and comprised the final analytic sample. To evaluate attrition from M2 to M3 (i.e., an important source of attrition bias), we compared individuals who completed all three substudies with those who were eligible at M2 but dropped out at M3. Attrition analyses are presented in Supplementary Tables 1 and 2.

At baseline, participants were in mid-to-late adulthood (*M *= 55.48, *SD *= 11.24), of whom 55.6% were women and 44.4% were men. The baseline mean household income was USD $80,707 (*SD* = $63,028). Regarding race, 95.0% were White, whereas the remaining were non-White (Multiracial, African American or Black, Native American or Aleutian Islander/Eskimo, Asian or Pacific Islander, Other, or did not disclose). Regarding education, 40.8% had a four-year college education or higher, whereas the remaining 59.2% did not disclose, had no high school degree, completed high school, or had some college.

### Procedures

At baseline, participants underwent the M2 Daily Stress Project procedures, which included 4 days of four-times-daily salivary cortisol sampling and separately completed an 8-day daily diary interview (Almeida et al., 2025). Subsequently, they underwent a telephone-delivered neurocognition test. Approximately 9 years later (M3), individuals completed a similar telephone-delivered neurocognition test to produce the prospective EF outcome.

### Measures

#### Baseline and 9-year EF

EF was measured at both baseline (M2) and a 9-year follow-up (M3) using the Brief Test of Adult Cognition by Telephone (BTACT), a computer-assisted, phone-administered test that takes about 15–20 min (Lachman et al., 2014). The BTACT comprises seven subtests, five of which were used here, each assessing a distinct EF dimension: (1) Backward Digit Span (working memory [WM]: reiterating alphanumeric strings in reverse sequence); (2) Category Fluency (semantic fluency: verbally stating objects within 60 s); (3) Stop-and-Go Switch Task (SGST) (inhibition and set-shifting); (4) Number Series (inductive reasoning: determining the following digit in a sequence); and (5) Backward Counting (processing speed and sustained attention: counting backwards within 30 s). Previous research has shown that the BTACT has excellent internal consistency, good retest reliability, and construct validity (Lachman et al., 2014). Higher scores denoted better overall EF capacity.

Across all models, the 9-year EF composite score was defined as the outcome, and baseline EF was consistently a covariate. This method models the residualized change in 9-year EF (i.e., the proportion of variance in 9-year EF unexplained by baseline EF), thereby evaluating how baseline cortisol, stressor, and worry severity predicted the longitudinal course of EF. Each subtest was standardized based on wave-specific mean and standard deviation scores (Supplementary Table 3), and mean subtest scores were computed to create a composite *z*-score modeling WM, semantic fluency, inhibition, set-shifting, inductive reasoning, and processing speed. Supplementary Tables 3–4 present the subtest standardization terms and the EF composite descriptive statistics by time point.

#### Baseline cortisol dysregulation

Cortisol samples were collected at home using a standardized kit with 16 color-coded, numbered Salivette tubes and detailed instructions (Sarstedt, Nümbrecht, Germany; Almeida et al., 2025). MIDUS staff verbally reviewed the data collection steps with each participant the next morning. Over four consecutive days, participants collected saliva at four time points: (1) immediately after waking; (2) 30 min post-awakening; (3) before lunch; and (4) before bedtime. To avoid contamination, participants refrained from caffeine, eating, or toothbrushing for 30 min before each collection. After the 4-day period, participants returned their Salivette tubes to a MIDUS lab, where samples were stored at −60°C prior to assay (Almeida et al., 2009). Protocol adherence was confirmed through quality checks of data-collection timing before the assay procedures.

We computed cortisol AUC using the trapezoidal rule on raw cortisol levels (Karlamangla et al., 2019). The formula (AUC_G_ = Σ[(m_(i+1)_ + m_i_) × t_i_]/2) sums trapezoids formed across each sample interval, with m_i_ as cortisol at each time point and t_i_ as the interval between samples (Olivera-Figueroa et al., 2015). Total AUC_G_, reflecting overall cortisol exposure, was calculated over the first 16 hr post-awakening; higher values indicate greater output (Olivera-Figueroa et al., 2015). CAR was computed by subtracting the cortisol value immediately after awakening from the value 30 min later. Lower or negative CAR values represent a blunted response. Diurnal cortisol slopes were calculated by subtracting post-awakening cortisol from pre-bedtime cortisol (log-transformed), then dividing by hours elapsed. More negative slopes indicate steeper cortisol declines, while values near zero or positive suggest flatter (dysregulated) slopes. Over the 4-day sampling timeframe, the mean of each metric was computed across days to yield an individual-level average score for all analyses.

#### Baseline daily stressor severity

Daily stressor severity was measured using the National Survey of Daily Experiences (NSDE; Almeida et al., 2009), a semi-structured daily diary phone interview conducted over eight consecutive evenings. Each evening, individuals indicated whether a stressor emerged across seven categories (Arguments/Disagreements, Avoided Arguments, Work/School Stress, Home Stress, Discrimination, Network Stress, and Other) and, if present, rated its severity (Almeida et al., 2009). Daily stressor severity was rated on a 4-point Likert scale (0 = *not at all stressful* to 3 = *very stressful*). On stressor-free days, severity was assigned a value of 0. Within each diary day, mean severity ratings were computed across all stressor categories that emerged (see Supplementary Tables 5–6 for source variable and category-level details). The NSDE has shown high internal consistency and strong construct validity (Almeida et al., 2009). Distributional details of daily stressor severity by number of simultaneous stressors are provided in Supplementary Table 7. In the present sample, variance decomposition indicated that daily stressor severity was primarily a within-person variable (intra-class correlation [ICC] = 0.268), with 73.2% attributable to day-to-day variation rather than stable trait-level differences (Supplementary Tables 8–10). Therefore, daily stressor severity is treated throughout as a day-level moderating factor in modeling perceived stress exposure, rather than as a stable trait-level variable.

#### Baseline GAD severity (chronic worry severity)

Chronic worry severity was measured continuously as a dimensional score rather than a binary categorical diagnosis by using the GAD module of the Composite International Diagnostic Interview-Short Form (CIDI-SF; Kessler et al., 1998). The CIDI-SF GAD module was consistent with the Diagnostic and Statistical Manual of Mental Disorders (DSM), 3rd Edition-Revised (DSM-III-R; Kessler et al., 1998). Although the DSM-III-R criteria informed the construction of the CIDI-SF, the primary GAD symptom content (e.g., excessive worry, irritability, muscle tension, restlessness, sleep disturbances) largely overlaps with the DSM 5th Edition (DSM-5) criteria (American Psychiatric Association, 2013; Brown et al., 1995), supporting its sustained construct validity. The measured symptoms necessitated worry on most days, thereby capturing persistent rather than episodic worry and indicating a trait-level, chronic aspect of anxiety. For each GAD symptom (e.g., concentration issues, fatigue), participants rated the severity on a 4-point Likert scale (1 = *never* to 4 = *on most days*). The measure showed good internal consistency (Cronbach’s α and McDonald’s ω ≥ 0.96 across both time points) and strong construct validity (Ng et al., 2024). Moreover, retest reliability (ICC = 0.423) reflected moderate temporal stability across both waves (Supplementary Tables 11–13).

### Data analysis

All data preprocessing and analytic steps were conducted in *R* (version 4.4.1). Specifically, the *dplyr* and *ggplot2* packages were used for data organization and visualization, while the *mgcv* and *lme4* packages were used for model estimation. All continuous predictors were standardized to *z*-scores, improving the interpretation of effect sizes and model convergence. The final analytic sample included 1,617 individuals. Pre-imputation subtest missingness ranged from 9.6% to 11.9% at baseline and from 25.1% to 26.8% at follow-up (Supplementary Table 2). Collectively, missingness was 18.1%, thus requiring an imputation approach. Random forest imputation was used to handle missing data, given its advantages over standard multiple imputation with chained equations or complete-case analysis, such as its ability to accommodate interactions and nonlinearities (Shah et al., 2014; Tang & Ishwaran, 2017).

The linear mixed models (LMMs) modeled within-person change over two waves, with time coded as a binary indicator (0 = M2 baseline; 1 = M3 follow-up), and repeated measures were nested within individuals. Conditional and marginal *R*² were calculated (Nakagawa et al., 2013). The unconditional ICCs were computed from a null model to estimate baseline between-person nesting (Supplementary Tables 14–15). For generalized additive mixed models (GAMMs), deviance explained was reported instead of *R*² and ICCs (Wood, 2017). Unlike LMMs, which presume linear predictor–outcome associations, GAMMs quantify smooth, possibly nonlinear functional forms using spline-derived basis functions. For interaction terms in particular, tensor product smooths allow the integrated effect of two predictors to vary in versatile ways across their joint range, rather than remaining constant. For each of the three unique cortisol metrics, seven models were evaluated: Model 1 (covariates and time); Model 2 (+ cortisol metric and stressor/worry as main effects); Model 3 (+ three-way cortisol × stressor/worry × time interaction, assessing study hypotheses); Model 4 (random slopes for stressor/worry); Model 5 (random slopes for cortisol metric); Model 6 (GAMMs with tensor product interaction smooth [*ti*]); and Model 7 (GAMM with full tensor product smooth [*te*] (Supplementary Tables 16–17) (Wood, 2017). The sequence indicates *a priori*, theory-based steps instead of an arbitrarily specified set of models. Each step builds sequentially from covariate control to fixed-effect moderation to individual-specific heterogeneity via random slopes, and finally to relaxed linear assumptions via GAMMs. To compare nested models, we assessed model fit using the Akaike Information Criterion (AIC) and the Bayesian Information Criterion (BIC; lower values indicate better fit; Burnham & Anderson, 2004). Changes in AIC and BIC (ΔAIC and ΔBIC) reflected changes compared to the best-fitting model.

To adjust for potential confounders, all models controlled for the following M2 baseline covariates: age, self-reported sex, baseline EF, thyroid disease status, counts of chronic illnesses, medication counts, medication use status, sleep quality, body mass index (BMI), and smoking status. Since sleep quality, smoking status, and body mass index may partially mediate the effects of cortisol on long-term EF, sensitivity analyses excluding these variables were conducted (Supplementary Tables 18–19). Cohen’s *d* effect sizes and their 95% confidence intervals (CIs) were computed and reported for fixed-effect size estimates (Feingold, 2015). Multiple testing was adjusted using the Benjamini–Hochberg false discovery rate (FDR) correction (Benjamini & Hochberg, 1995). For any significant omnibus interaction terms, we estimated simple slopes at low (−1 *SD*), average (0 *SD*), and high (+1 *SD*) moderator levels, indicating specific raw-score probe values rather than range cut-points (Supplementary Table 20). For any non-significant omnibus interaction, simple slope analyses were performed as pre-specified exploratory analyses. All random-effects models included random intercepts for each participant; optimal models also included random slopes for the stressor/worry variable (Model 4), aligned with guidelines for daily diary approaches (Laurenceau et al., 2026). Finally, key model equations are detailed in Supplementary Table 23.

## Results

### Model selection and variance decomposition

Across all models, Model 4 offered the optimal fit (Supplementary Tables 16 and 17). For daily stressor severity moderator models, Model 4 had considerably better fit than other options across all three cortisol metrics (ΔAIC range: 691.3–723.9 [AUC]; 688.7–727.4 [CAR]; 699.9–723.0 [DCS]). This model explained a large proportion of the variance (>95%) in 9-year EF (conditional *R*²). The conditional ICC from Model 4 suggested that about 77% of the residual variance was attributed to between-person trait-level differences (Supplementary Tables 14–15). Comparatively, residual within-person variance was small (ICC = 4.0%; variance = 2.0%). The intercept-slope covariance was uniformly negative across models (range: −0.143 to −0.145; ICC range: −28.1% to −28.6%), suggesting that individuals with higher EF typically exhibited more negative stressor/worry–EF slopes (Supplementary Table 21). In addition, sensitivity analyses comparing binary vs. 9-year elapsed-time coding confirmed stable main effects, but the time × stressor severity interaction was statistically significant only under binary coding (Supplementary Table 22). As detailed below, variations in the three-way interaction terms emerged depending on the cortisol metric or the stressor/worry type.

### Daily stressor severity as a moderator of cortisol dysregulation and 9-year EF (hypothesis 1)

Table 1 summarizes fixed-effects estimates from the best-fitting model. Greater daily stressor severity was significantly related with higher 9-year EF scores (AUC: *d *= 0.141, 95% CI [0.044, 0.244], *p*[FDR] = .010; CAR: *d *= 0.143, 95% CI [0.046, 0.246], *p*[FDR] = .008; DCS: *d *= 0.135, 95% CI [0.036, 0.228], *p*[FDR] = .012). Neither AUC (*d *= −0.088, 95% CI [−0.187, 0.010], *p*(FDR) = .099) nor CAR (*d *= −0.060, 95% CI [−0.158, 0.037], *p*(FDR) = .290) significantly interacted with this stressor–EF pathway. Nonetheless, daily stressor severity significantly interacted with DCS to forecast 9-year EF scores (adjusting for baseline EF) (*d *= 0.113, 95% CI [0.017, 0.214], *p*[FDR] = .033). Main-effect estimates were stable before and after the addition of the interaction term (Table 4). Figure 1A and Table 3 detail this interaction. Flatter DCS values were significantly linked to poorer EF at all three levels of daily stressor severity: low (*d *= −0.143, 95% CI [−0.241, −0.045], *p* = .004), mean (*d *= −0.126, 95% CI [−0.224, −0.029], *p* = .011), and high (*d *= −0.106, 95% CI [−0.204, −0.008], *p* = .033). Despite the DCS-EF relationship attenuating at higher stressor severity, the 95% CIs across severity levels overlapped substantially, and formal difference tests were not statistically significant (Supplementary Tables 20 and 24). This weaker pattern should thus be regarded as exploratory instead of conclusive.

**Table 1:** Fixed-effects estimates for cortisol dysregulation metrics as moderators of the within-person association between daily stressor severity and 9-year executive functioning.

Metric	Term	Cohen’s *d*	95% CI LL	95% CI UL	*p* (Raw)	*p* (FDR)
(A) AUC	Intercept	0.020	–0.078	0.118	.692	.692
	AUC	–0.024	–0.122	0.074	.630	.692
	Daily stressor severity	0.141	0.044	0.244	.005	.010
	Age	–0.800	–0.897	–0.702	<.001	<.001
	Female	–0.125	–0.223	–0.027	.013	.020
	Executive functioning (baseline)	0.020	–0.078	0.119	.686	.692
	Thyroid disease	0.107	0.009	0.204	.033	.046
	Chronic illness	–0.294	–0.390	–0.195	<.001	<.001
	Number of medications	–0.136	–0.233	–0.038	.006	.011
	Medication use	–0.468	–0.565	–0.369	<.001	<.001
	Sleep quality	–0.321	–0.416	–0.222	<.001	<.001
	Smoking status	0.349	0.252	0.449	<.001	<.001
	Body mass index	–0.463	–0.559	–0.364	<.001	<.001
	AUC × Daily stress severity	–0.088	–0.187	0.010	.078	.099
(B) CAR	Intercept	0.020	–0.078	0.118	.688	.688
	CAR	0.020	–0.078	0.118	.687	.688
	Daily stressor severity	0.143	0.046	0.246	.004	.008
	Age	–0.813	–0.911	–0.715	<.001	<.001
	Female	–0.125	–0.223	–0.027	.013	.020
	Executive functioning (baseline)	0.020	–0.078	0.119	.682	.688
	Thyroid disease	0.106	0.009	0.204	.033	.047
	Chronic illness	–0.294	–0.390	–0.195	<.001	<.001
	Number of medications	–0.138	–0.236	–0.040	.006	.010
	Medication use	–0.465	–0.562	–0.366	<.001	<.001
	Sleep quality	–0.321	–0.416	–0.222	<.001	<.001
	Smoking status	0.349	0.252	0.449	<.001	<.001
	Body mass index	–0.464	–0.561	–0.365	<.001	<.001
	CAR × Daily stress severity	–0.060	–0.158	0.037	.228	.290
(C) DCS	Intercept	0.009	–0.089	0.107	.859	.859
	DCS	–0.126	–0.224	–0.029	.012	.018
	Daily stressor severity	0.135	0.036	0.228	.007	.012
	Age	–0.824	–0.924	–0.728	.000	.000
	Female	–0.110	–0.208	–0.012	.028	.036
	Executive functioning (baseline)	0.022	–0.076	0.120	.664	.715
	Thyroid disease	0.107	0.010	0.204	.032	.038
	Chronic illness	–0.293	–0.389	–0.194	<.001	<.001
	Number of medications	–0.135	–0.232	–0.036	.007	.012
	Medication use	–0.465	–0.562	–0.367	<.001	<.001
	Sleep quality	–0.321	–0.416	–0.222	<.001	<.001
	Smoking status	0.348	0.250	0.447	<.001	<.001
	Body mass index	–0.454	–0.551	–0.356	<.001	<.001
	DCS × Daily stress severity	0.113	0.017	0.214	.023	.033

*Note*. AUC = total area under the cortisol curve with respect to ground; CAR = cortisol awakening response; CI = confidence interval; DCS = diurnal cortisol slope; *d* = standardized effect size; LL = 95% CI lower level; *p*(Raw) = unadjusted two-tailed *p*-value; *p*(FDR) = *p*-value adjusted for multiple comparisons using the Benjamini–Hochberg false discovery rate correction applied across all variables within each panel; UL = 95% CI upper level. Each panel presents fixed-effects estimates from the best-fitting linear mixed model (M4: Random Slopes [Worry]; see Supplementary Tables 16–17) for each cortisol metric. All models were estimated using restricted maximum likelihood (REML) with random intercepts and random slopes for chronic worry severity.

**Figure 1:**
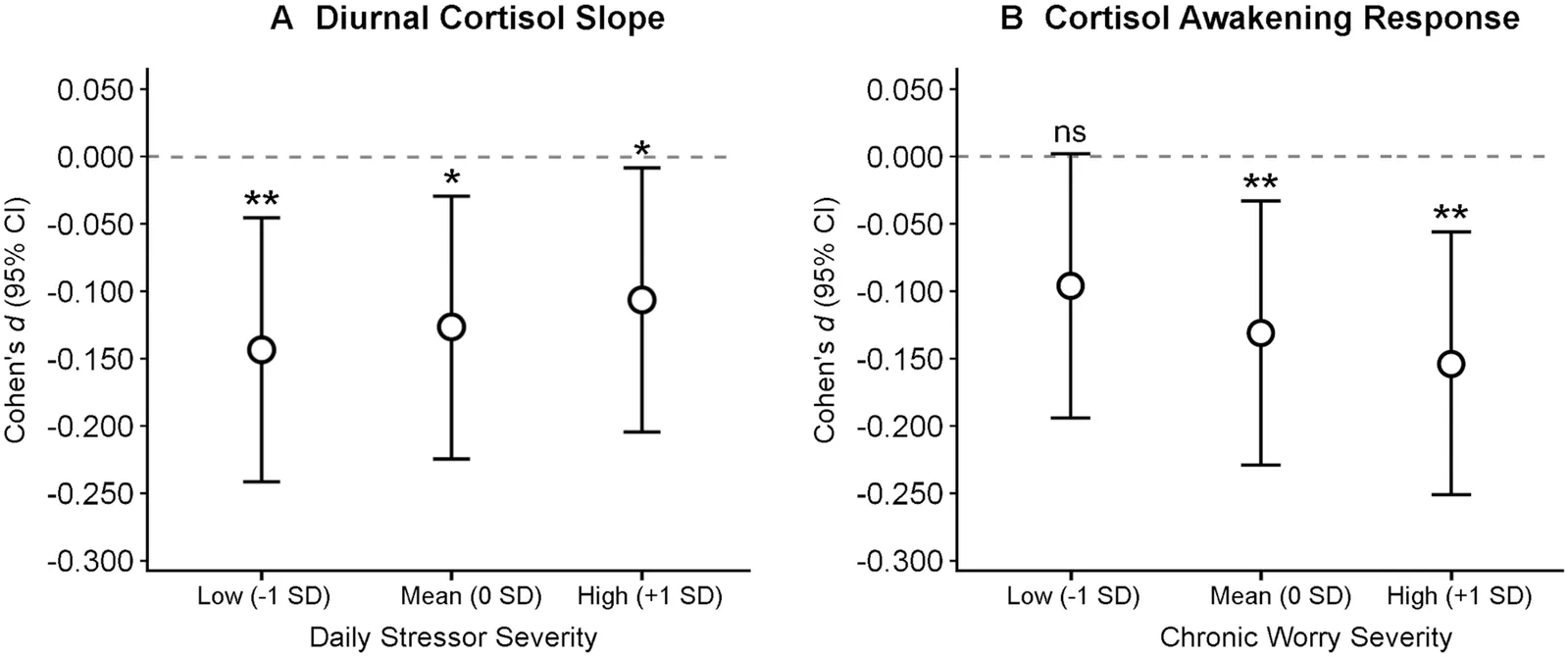
Simple slopes of DCS and CAR on 9-year executive functioning by daily stressor or chronic worry severity level. CAR = cortisol awakening response; CI = confidence interval; ns = nonsignificant; DCS = diurnal cortisol slope; FDR = false discovery rate. Simple slopes were estimated from the best-fitting linear mixed model (model 4: Random slopes [stressor] in panel A; random slopes [worry] in panel B), reflecting the effect of each cortisol metric on executive functioning at low, mean, and high levels of daily stressor severity (A) or chronic worry severity (B). Open circles represent point estimates (Cohen’s *d*); vertical bars represent 95% CIs. The dashed horizontal line indicates *d *= 0 (no effect). For DCS, all three simple slopes were statistically significant. For CAR, none of the simple slopes reached statistical significance at low (*p* = .055), mean (*p* = .808), or high (*p* = .149) chronic worry severity. *p <.05. **p <.01.

### Chronic worry severity as a moderator of cortisol dysregulation and 9-year EF (hypothesis 2)

Table 2 presents fixed-effects estimates from the best-fitting model. Across all three cortisol metrics, higher chronic worry severity was significantly related to lower 9-year EF scores (adjusting for baseline EF; AUC: *d *= −0.290, 95% CI [−0.387, −0.192], *p*[FDR] <.001; CAR: *d *= −0.367, 95% CI [−0.468, −0.271], *p*[FDR] <.001; DCS: *d *= −0.216, 95% CI [−0.316, −0.119], *p*[FDR] <.001). Chronic worry severity did not significantly interact with AUC (*d *= 0.008, 95% CI [−0.090, 0.106], *p*(FDR) = .875) or DCS (*d *= −0.090, 95% CI [−0.186, 0.008], *p*(FDR) = .084) to predict 9-year EF. However, the severity of chronic worry significantly moderated the CAR-to-EF relationship (*d *= −0.176, 95% CI [−0.276, −0.079], *p*[FDR] = .001; Figure 1B). Simple slope analyses suggested that CAR was not significantly related to 9-year EF at any chronic worry severity level (low: *d *= 0.096, 95% CI [−0.002, 0.193], *p* = .055; mean: *d = *0.012, 95% CI [−0.086, 0.110], *p* = .808; high: *d *= −0.072, 95% CI [−0.170, 0.026], *p* = .149; Table 3). Additionally, despite the non-significantly DCS × chronic worry severity omnibus interaction, exploratory simple slope analyses indicated that flatter DCS was related to poorer 9-year EF at average and high, but not low, worry severity levels (low: *d *= −0.096, 95% CI [−0.194, 0.002], *p* = .054; mean: *d *= −0.131, 95% CI [−0.229, −0.033], *p* = .009; high: *d *= −0.154, 95% CI [−0.251, −0.056], *p* = .002; Table 3). Such exploratory findings require independent replication.

**Table 2:** Fixed-effects estimates for cortisol dysregulation metrics as moderators of the within-person association between chronic worry severity and 9-year executive functioning.

Metric	Terms	Cohen’s *d*	95% CI LL	95% CI UL	*p* (Raw)	*p* (FDR)
(A) AUC	Intercept	0.024	–0.074	0.121	.634	.743
	AUC	–0.022	–0.120	0.076	.653	.743
	Chronic worry severity	–0.290	–0.387	–0.192	<.001	<.001
	Age	–0.830	–0.925	–0.730	<.001	<.001
	Female	–0.129	–0.227	–0.031	.010	.014
	Executive functioning (baseline)	0.020	–0.078	0.118	.690	.743
	Thyroid disease	0.157	0.060	0.257	.002	.003
	Chronic illness	–0.240	–0.338	–0.143	<.001	<.001
	Number of medications	–0.180	–0.279	–0.083	<.001	.001
	Prescribed medication	–0.420	–0.516	–0.321	<.001	<.001
	Sleep quality	–0.275	–0.377	–0.179	<.001	<.001
	Smoking status	0.347	0.250	0.446	<.001	<.001
	Body mass index	–0.384	–0.485	–0.288	<.001	<.001
	AUC × Chronic Worry Severity	0.008	–0.090	0.106	.875	.875
(B) CAR	Intercept	0.019	–0.079	0.116	.709	.764
	CAR	0.012	–0.086	0.110	.808	.808
	Chronic worry severity	–0.367	–0.468	–0.271	<.001	<.001
	Age	–0.845	–0.942	–0.747	<.001	<.001
	Female	–0.126	–0.224	–0.029	.012	.015
	Executive functioning (baseline)	0.020	–0.078	0.118	.688	.764
	Thyroid disease	0.150	0.053	0.250	.003	.004
	Chronic illness	–0.241	–0.341	–0.144	<.001	<.001
	Number of medications	–0.184	–0.282	–0.086	<.001	<.001
	Medication use	–0.416	–0.515	–0.319	<.001	<.001
	Sleep quality	–0.271	–0.371	–0.174	<.001	<.001
	Smoking status	0.346	0.249	0.444	<.001	<.001
	Body mass index	–0.386	–0.485	–0.290	<.001	<.001
	CAR × Chronic worry severity	–0.176	–0.276	–0.079	<.001	.001
(C) DCS	Intercept	0.013	–0.085	0.111	.800	.800
	DCS	–0.131	–0.229	–0.033	.009	.012
	Chronic worry severity	–0.216	–0.316	–0.119	<.001	<.001
	Age	–0.853	–0.951	–0.755	<.001	<.001
	Female	–0.113	–0.211	–0.015	.024	.030
	Executive functioning (baseline)	0.021	–0.077	0.120	.668	.719
	Thyroid disease	0.156	0.058	0.256	.002	.003
	Chronic illness	–0.243	–0.340	–0.144	<.001	<.001
	Number of medications	–0.180	–0.279	–0.082	<.001	.001
	Medication use	–0.417	–0.516	–0.320	<.001	<.001
	Sleep quality	–0.274	–0.369	–0.175	<.001	<.001
	Smoking status	0.349	0.251	0.447	<.001	<.001
	Body mass index	–0.380	–0.480	–0.283	<.001	<.001
	DCS × Chronic worry severity	–0.090	–0.186	0.008	.072	.084

*Note*. AUC = total area under the cortisol curve with respect to ground; CAR = cortisol awakening response; CI = confidence interval; DCS = diurnal cortisol slope; *d* = standardized effect size; LL = 95% CI lower level; *p*(Raw) = unadjusted two-tailed *p*-value; *p*(FDR) = *p*-value adjusted for multiple comparisons using the Benjamini–Hochberg false discovery rate correction applied across all variables within each panel; UL = 95% CI upper level. Each panel presents fixed-effects estimates from the best-fitting linear mixed model (M4: Random Slopes [Stressor]; see Supplementary Tables 16–17) for each cortisol metric. All models were estimated using restricted maximum likelihood (REML) with random intercepts and random slopes for chronic worry severity.

**Table 3:** Simple slopes of the cortisol dysregulation metric × daily stressor/chronic worry severity interaction on 9-year executive functioning by daily stressor/chronic worry severity level.

Variables	Cohen’s *d*	95% CI LL	95% CI UL	*p*
**(A) AUC × daily stress severity**				
Low (–1 *SD*)	–0.010	–0.108	0.088	.847
Mean (0 *SD*)	–0.024	–0.122	0.074	.630
High (+1 *SD*)	–0.038	–0.136	0.060	.447
**(B) CAR × daily stress severity**				
Low (–1 *SD*)	0.030	–0.069	0.128	.554
Mean (0 *SD*)	0.020	–0.078	0.118	.687
High (+1 *SD*)	0.010	–0.088	0.108	.839
**(C) DCS × daily stress severity**				
Low (–1 *SD*)	–0.143	–0.241	–0.045	.004
Mean (0 *SD*)	–0.126	–0.224	–0.029	.011
High (+1 *SD*)	–0.106	–0.204	–0.008	.033
**(D) AUC × chronic worry severity**				
Low (–1 *SD*)	–0.024	–0.122	0.074	.634
Mean (0 *SD*)	–0.022	–0.120	0.076	.653
High (+1 *SD*)	–0.019	–0.116	0.079	.709
**(E) CAR × chronic worry severity**				
Low (–1 *SD*)	0.096	–0.002	0.193	.055
Mean (0 *SD*)	0.012	–0.086	0.110	.808
High (+1 *SD*)	–0.072	–0.170	0.026	.149
**(F) DCS × chronic worry severity**				
Low (–1 *SD*)	–0.096	–0.194	0.002	.054
Mean (0 *SD*)	–0.131	–0.229	–0.033	.009
High (+1 *SD*)	–0.154	–0.251	–0.056	.002

*Note*. AUC = total area under the cortisol curve with respect to ground; CAR = cortisol awakening response; DCS = diurnal cortisol slope; CI = confidence interval; LL = 95% CI lower level; UL = 95% CI upper level. Cohen’s *d* = standardized effect size. Simple slopes were derived from the best-fitting model (M4: Random Slopes [Stressor/Worry]; see Supplementary Tables 16–17) by estimating the effect of each cortisol metric on executive functioning at low (−1 *SD*), mean (0 *SD*), and high (+1 *SD*) levels of daily stressor/chronic worry severity.

**Table 4:** Summary of fixed-effects estimate changes from model 2 (main effects) to model 3 (primary model) by cortisol metric.

Parameter	*b* (Model 2)	*b* (Model 3)	Δ |*b*|	Δ*b*%	*p* (Model 2)	*p* (Model 3)
**(A) Cortisol awakening response**						
Intercept	0.003	**0.003**	0.000	10.7	.899	.891
Time	0.000	−0.001	0.000	−133.3	.980	.956
z (Slope mean)	−0.045	−0.044	0.001	2.5	.004	.006
z (Stressor severity)	−0.053	−0.039	0.014	26.9	.000	.000
z (Age)	−0.214	−0.213	0.001	0.3	.000	.000
Women vs. Men	−0.052	−0.050	0.002	3.1	.065	.074
z (Global cognition)	0.004	0.004	0.000	0	.760	.762
z (Self-rated health)	0.012	0.012	0.000	−2.5	.000	.000
z (Chronic illnesses)	−0.090	−0.092	−0.002	−1.8	.000	.000
z (Prescription medications)	−0.005	−0.005	0.000	4.3	.443	.462
z (Non-prescription medications)	−0.126	−0.126	0.000	0.1	.000	.000
Sleep disturbances	−0.018	−0.019	0.000	−2.2	.000	.000
Smoking status	0.053	0.054	0.000	0.6	.000	.000
z (Body mass index)	−0.052	−0.053	−0.001	−1.5	.000	.000
Time × z (Slope mean)	0.010	0.010	0.000	−3	.429	.442
Time × z (Stressor severity)	0.024	0.020	−0.004	−15.5	.000	.008
**(B) Diurnal cortisol slope**						
Intercept	0.003	0.003	0.000	10.7	.899	.891
Time	0.000	−0.001	0.000	−133.3	.980	.956
z (Slope mean)	−0.045	−0.044	0.001	2.5	.004	.006
z (Stressor severity)	−0.053	−0.039	0.014	26.9	.000	.000
z (Age)	−0.214	−0.213	0.001	0.3	.000	.000
Women vs. Men	−0.052	−0.050	0.002	3.1	.065	.074
z (Global cognition)	0.004	0.004	0.000	0	.760	.762
z (Self-rated health)	0.012	0.012	0.000	−2.5	.000	.000
z (Chronic illnesses)	−0.090	−0.092	−0.002	−1.8	.000	.000
z (Prescription medications)	−0.005	−0.005	0.000	4.3	.443	.462
z (Non-prescription medications)	−0.126	−0.126	0.000	0.1	.000	.000
Sleep disturbances	−0.018	−0.019	0.000	−2.2	.000	.000
Smoking status	0.053	0.054	0.000	0.6	.000	.000
z (Body mass index)	−0.052	−0.053	−0.001	−1.5	.000	.000
Time × z (Slope mean)	0.010	0.010	0.000	−3	.429	.442
Time × z (Stressor severity)	0.024	0.020	−0.004	−15.5	.000	.008
**(C) Total cortisol area under the curve**						
Intercept	0.008	0.009	0.001	8.6	.718	.694
Time	0.000	−0.001	−0.001	−200	.981	.942
z (Slope mean)	0.001	0.001	0.000	18.2	.944	.935
z (Stressor severity)	−0.053	−0.045	0.007	14.1	.000	.000
z (Age)	−0.211	−0.211	0.000	0	.000	.000
Women vs. Men	−0.061	−0.061	0.000	0	.030	.030
z (Global cognition)	0.004	0.004	0.000	−2.6	.785	.787
z (Self-rated health)	0.012	0.012	0.000	0.8	.000	.000
z (Chronic illnesses)	−0.091	−0.089	0.001	1.4	.000	.000
z (Prescription medications)	−0.005	−0.004	0.000	6.4	.442	.475
z (Non-prescription medications)	−0.127	−0.128	−0.001	−0.9	.000	.000
Sleep disturbances	−0.018	−0.019	0.000	−1.1	.000	.000
Smoking status	0.053	0.053	0.000	0	.000	.000
z (Body mass index)	−0.052	−0.053	0.000	−0.8	.000	.000
Time × z (Slope mean)	−0.016	−0.016	0.000	−1.3	.209	.206
Time × z (Stressor severity)	0.024	0.018	−0.006	−25.3	.000	.012

*Note*. *b* = unstandardized regression estimate.

## Discussion

This prospective study investigated whether baseline cortisol dysregulation was associated with residualized 9-year EF, and whether daily stressor severity or chronic worry severity moderated this relationship, among midlife and older community adults. After FDR correction, two omnibus interactions were statistically significant. Greater severity of daily stressors moderated the relationship between flatter DCS and lower residualized 9-year EF, and greater chronic worry severity moderated the CAR-to-EF relationship. Meaning, flatter DCS was associated with worse EF across all severity levels, but the observed weaker trend of this relationship at higher severity levels should be interpreted with caution due to overlap in the CIs and the statistically non-significant formal difference tests. Moreover, exploratory simple-slopes analyses indicated that flatter DCS was associated with poorer residualized 9-year EF at average and high levels of worry severity. Nonetheless, the DCS × worry omnibus interaction was non-significant after FDR correction and therefore requires replication.

The uniqueness of DCS among all three cortisol metrics in interacting with daily stressor severity is theoretically cogent. Unlike AUC—which reflects cortisol exposure buildup (Golden et al., 2013)—or CAR—which indicates acute HPA sensitivity (Clow et al., 2010)—DCS marks the sleep-wake circadian regulatory cycle of the HPA axis modulated by the suprachiasmatic nucleus (SCN; O’Byrne et al., 2021). Blunted DCS has been linked to disturbances in the sleep–wake circadian modulatory feedback loop (Tsui et al., 2020) and to modified glucocorticoid receptor responses in the hippocampus and prefrontal cortex (PFC; Su et al., 2025). These neural structures are integral to inhibition, set shifting, and WM (Girotti et al., 2018), which are measured by the BTACT. This mediation path reflects a key direction in which daily stressor exposure or chronic worry operates via a flatter DCS to affect long-term EF was not examined herein, since cortisol was assessed at the baseline occasion and longitudinal cortisol data were unavailable at the time of data retrieval. The reverse direction reflects a key direction for future prospective studies with repeated cortisol measurements.

Although the strength of the DCS–EF relationship was observed to weaken at higher levels of daily stressor severity, this trend was not statistically supported, given overlapping CIs and non-significant formal difference tests across severity levels. Thus, this outcome should be carefully interpreted. This provisional trend, if replicated, might suggest that EF demands are already linked to high daily stress exposure (Girotti et al., 2024; Li et al., 2025), which could reduce the observed incremental predictive value of DCS at higher daily stress severity. However, blunted DCS was significantly associated with poorer residualized 9-year EF across all three stressor severity levels, implying that flatter daily cortisol trends confer correlational importance for long-term EF irrespective of concurrent daily stress exposure.

In exploratory analyses, the trend whereby blunted DCS was associated with poorer 9-year EF at average and high, but not low, chronic worry severity is provisionally aligned with the biological sensitivity framework (Ellis et al., 2011). However, this viewpoint necessitates replication given the non-significant omnibus interaction. Chronic worry has been linked to chronic HPA hypersensitivity (Tafet & Nemeroff, 2020), modified hippocampal neurogenesis and glucocorticoid receptor re-sensitization (Odaka et al., 2017), and decreased PFC-related HPA axis inhibition (Sullivan & Gratton, 2002). These paths can help explain why greater chronic worry severity could magnify the relationship between flatter DCS and long-term EF, but this mechanism requires direct empirical investigation.

The current findings build on previous research in several ways. An individual-participant data meta-analysis of five cohorts showed that flatter DCS was associated with weaker fluid reasoning (Gardner et al., 2019), consistent with the current DCS simple slopes. Another study found that greater blunting of DCS was associated with declines in global cognitive functioning and verbal fluency over 5–9 years, even after adjusting for baseline neurocognitive performance (Tsui et al., 2020). The current study parallels this analytic method by modeling residualized 9-year EF with baseline EF as a covariate. Rickenbach et al. (2014) observed that daily stress magnified the relationship between blunted CAR and declining memory over nine years, providing a partial basis for a stress-moderated cortisol-to-EF link, but the cortisol metric and cognitive outcome differ from those herein. The null moderation outcomes for AUC with both moderators and for CAR with daily stressor severity are broadly consistent with previous research (Segerstrom et al., 2016). Plausibly, global cortisol exposure may necessitate longer follow-up intervals or older samples to observe identifiable relationships with EF. On that note, the CIDI-SF GAD module was used as a continuous index of chronic worry severity rather than a binary diagnostic threshold. The moderate 9-year retest reliability of this composite (ICC = 42.3%) supports its use as a temporally stable index of chronic worry, consistent with the broader definition of GAD as dimensional rather than categorical (Newman et al., 2013).

Some limitations merit consideration. First, cortisol, stressor severity, and worry severity were each assessed solely at baseline, hindering conclusions about the temporal buildup and intensification of HPA-axis dysregulation over the 9-year period. Second, despite adjusting for several M2 baseline covariates (e.g., BMI, sleep quality, smoking status), which can partially mediate rather than solely confound cortisol effects on EF, bias from unmeasured confounders remains given the observational design. Our sensitivity analyses, which excluded these possible mediators, generated similar findings (Supplementary Tables 18–19). Third, about 18% of the data required random forest imputation, and in those cases, the MAR assumption could not be tested. The final analytic sample comprised about one-third of the broader MIDUS cohort due to the multi-substudy participation criteria. Relatedly, attrition analyses showed that included (vs. excluded) individuals were generally slightly older and more likely to be White (Supplementary Table S1), which constrains generalizability and warrants careful interpretation given selection bias. Fourth, reliance on a composite EF score aggregated across five EF-pertinent subdomains hindered domain-specific interaction tests that could reveal the differential risk of specific EF dimensions for cortisol dysregulation. Finally, given the mostly White and socioeconomically stable sample, the generalizability of these patterns to more culturally diverse samples may be limited.

However, the study’s strengths included the 4-day, four-times-daily cortisol sampling, which generated reliable participant-mean indices (Segerstrom et al., 2014), the 8-day daily diary approach, which modeled day-level stressor exposure, and the 9-year longitudinal follow-up. Other strengths included the systematic seven-model selection approach using LMMs as the main method and GAMMs as adjunctive sensitivity checks. In addition, FDR adjustment was applied to all omnibus fixed-effect estimates, and biomedically relevant covariates (e.g., chronic illness, medication use, thyroid disease status) were consistently included to enhance analytic robustness. Finally, small effect sizes are generally expected in large longitudinal cohorts with long follow-up periods, in which any unique baseline biological risk factor is anticipated to explain only a small proportion of the variance in long-term EF changes, which are marked by considerable between-person heterogeneity.

These outcomes should be interpreted in the context of the study’s observational design and the exploratory approach of multiple secondary outcomes, which collectively prevent strong causal or clinical inferences. Blunted DCS was related to poorer residualized 9-year EF across the total sample. Moreover, exploratory findings suggested that this relationship may be stronger among those with higher severity of chronic worry, but this trend requires replication before drawing strong clinical inferences. Since about 45% of dementia cases have been associated with changeable risk factors (Bruck et al., 2025; Ma’u et al., 2025), flatter diurnal cortisol slope strengths may merit attention as a plausible risk factor in future treatment research. Experimental data establishing directional paths is necessary prior to drawing inferences about prevention strategies. Relatedly, lifestyle approaches that enhance morning daylight exposure and address sleep–wake regularity (Guindon et al., 2024; Robertson-Dixon et al., 2023) are candidates for future treatment studies, possibly alongside empirically supported techniques to alleviate chronic worry. However, the preventive value of worry-centered cognitive-behavioral therapy remains to be determined (Rosnick et al., 2016). Finally, replication in more culturally diverse samples and the use of more causally intensive experimental designs are required before findings can be translated to clinical practice.

## Supplementary Material

gbag178_Supplementary_Data

## Data Availability

This study was not preregistered. The MIDUS data can be obtained through the Inter-university Consortium for Political and Social Research (ICPSR) repository: https://www.icpsr.umich.edu/web/ICPSR/series/203. The analytic code (*R* scripts) used for all preprocessing and model estimation steps will be made available upon reasonable request to the corresponding author. Study materials, including all measures used, are already publicly available through the MIDUS study infrastructure.
