# Supplementary material for: Blunted diurnal cortisol slope as a risk marker for 9-year executive function decline: amplification by daily stress and worry severity
Source: J Gerontol B Psychol Sci Soc Sci. 2026 Aug 31;81(11):gbag178. doi: 10.1093/geronb/gbag178 (PMC13652025; doi:10.1093/geronb/gbag178)
Supplement: Supplementary file 1 [file gbag178_supplementary_data.pdf]

***The Journals of Gerontology, Series B: Psychological Sciences and Social Sciences***
**Supplementary Material: Zainal & Noble. Blunted diurnal cortisol slope as a risk marker for 9-year executive function decline: Amplification by daily stress and worry severity.**

**Contents**

Table S1. *Attrition Analysis at Baseline (M2) and 9-Year Follow-Up (M3)* ....................2
Table S2. *Missingness Detail by Domain, Item Type, and Missingness Level* ....................3
Table S3. *EF Subtest Wave-Specific Standardization Parameters* ....................4
Table S4. *EF Composite Descriptive Statistics by Assessment Wave* ....................5
Table S5. *Operationalization of Daily Stressor Severity: Construction, Aggregation Logic, and Verification* ..6
Table S6. *Individual Stressor Domain Severity Items* ....................7
Table S7. *Daily Stressor Severity by Number of Stressors per Diary Day* ....................8
Table S8. *Intraclass Correlation Coefficient (ICC) for Daily Stressor Severity: Variance Decomposition from Unconditional Null Models* ....................9
Table S9. *Variance Component Estimates from the Unconditional Null Model for Daily Stressor Severity (Baseline)* ....................10
Table S10. *Descriptive Statistics for Daily Stressor Severity (Day-Level and Person-Level)* ....................11
Table S11. *Internal Consistency of the 10-Item GAD Symptom Scale Across MIDUS Waves* ....................12
Table S12. *GAD Item Descriptives at M2 Baseline (Baseline)* ....................13
Table S13. *Test–Retest Reliability of GAD Severity: M2 to M3 (9-Year Interval)* ....................14
Table S14. *Variance Component Estimates from the Unconditional Null Model for the EF Composite* ........15
Table S15. *Within-Model Conditional ICCs Across Models 1–5* ....................16
Table S16. *Model Comparison Statistics for LMMs and GAMMs Examining Cortisol Dysregulation Indices as Moderators of the Association Between Daily Stressor Severity and 9-Year Executive Functioning* 17
Table S17. *Model Comparison Statistics for LMMs and GAMMs Examining Cortisol Dysregulation Indices as Moderators of the Association Between Chronic Worry Severity and 9-Year Executive Functioning*18
Table S18. *Full Fixed-Effect Estimates: Primary vs. Sensitivity Model* ....................19
Table S19. *Model Fit: Primary vs. Sensitivity Model* ....................20
Table S20. *Raw-Score Probe Values and Sample Distribution of Moderator Variables at Low (−1 SD), Mean (0 SD), and High (+1 SD) Probing Levels* ....................21
Table S21. *Random Effects Variance Components from the Best-Fitting LMMs Examining Cortisol Dysregulation Indices as Moderators of the Within-Person Association Between Daily Stressor Severity and 9-Year Executive Functioning* ....................22
Table S22. *Empirical Verification: Binary Time (0/1) vs. Elapsed-Year (~0/9) Model Comparison–Fixed Effects* ....................23
Table S23. *Full Specification of Model 3 (CAR Example): Cortisol Awakening Response by Chronic Worry Severity by Time Interaction, Testing Hypothesis 2* ....................24
Table S24. *Formal Difference Tests Between Low (-1 SD) and High (+1 SD) Simple Slopes of Cortisol Dysregulation on 9-Year Executive Functioning, Across All Six Cortisol Metric x Moderator Models* ....25

**Table S1.** ***Attrition Analysis at Baseline (M2) and 9-Year Follow-Up (M3)***

| Variable | Not Included | Included | Inferential Statistics | | | |
|---|---|---|---|---|---|---|
| | | | Statistic | *p* | Cohen's *d* | V |
| Continuous Variables | *n* = 2024 | *n* = 1270 | | | | |
| Age at M2 baseline assessment, years | 54.07 (11.48) | 55.48 (11.23) | W = 1382128 | < .001 | 0.123 | |
| Baseline EF composite score (M2) | 0.18 (1.07) | 0.17 (0.91) | W = 1286892 | .950 | -0.004 | |
| Number of chronic conditions (M2) | 2.32 (2.42) | 2.27 (2.23) | W = 1285764 | .984 | -0.019 | |
| Any prescription medication use - count (M2) | 0.66 (0.47) | 0.64 (0.48) | W = 1268506 | .445 | -0.027 | |
| Sleep quality - PSQI global score (M2) | 5.96 (3.65) | 5.70 (3.32) | W = 1251522 | .202 | -0.076 | |
| Body mass index, kg/m² (M2) | 29.11 (6.09) | 28.89 (5.78) | W = 1267152 | .496 | -0.036 | |
| Household income, USD (M1) | 84300.40 (70923.45) | 80707.09 (63028.24) | W = 1289600 | .870 | -0.053 | |
| Chronic worry severity - GAD scale (M2) | 0.15 (0.94) | 0.10 (0.72) | W = 1276334 | .233 | -0.059 | |
| Categorical Variables | *n* = 2024 | *n* = 1270 | | | | |
| Sex - Female, *n* (%) | 1104 (54.5%) | 706 (55.6%) | $\chi^2$ = 0.344 | .557 | | 0.010 |
| Race - White, *n* (%) | 1870 (92.4%) | 1206 (95.0%) | $\chi^2$ = 8.336 | .004 | | 0.050 |
| Education - College or higher, *n* (%) | 776 (38.3%) | 518 (40.8%) | $\chi^2$ = 1.960 | .162 | | 0.024 |
| Thyroid disease - Yes, *n* (%) | 251 (12.4%) | 172 (13.5%) | $\chi^2$ = 0.909 | .340 | | 0.017 |
| Ever smoked regularly - Yes, *n* (%) | 941 (46.5%) | 562 (44.3%) | $\chi^2$ = 1.578 | .209 | | 0.022 |
| Any prescription medication - Yes, *n* (%) | 1330 (65.7%) | 818 (64.4%) | $\chi^2$ = 0.583 | .445 | | 0.013 |

*Note.* M2 = Midlife Development in the United States (MIDUS) Wave 2 (2004–2009); M3 = MIDUS Wave 3 (2013–2014); *d* = effect sizes for continuous variables; EF = executive functioning; PSQI = Pittsburgh Sleep Quality Index; USD = United States Dollar; GAD = generalized anxiety disorder.

**Table S2.** ***Missingness Detail by Domain, Item Type, and Missingness Level***

| Parent Scale | Item Variables | Stage Description | *N* Total | *N* Missing | % Missing |
|---|---|---|---|---|---|
| **Domain: Outcome; Item Type: EF Subtest Score; Missingness Level: Item-Level (Subtest)** | | | | | |
| EF Composite | M2 EF Digit Backward Scale | Pre-Imputation | 6479 | 624 | 9.63 |
| EF Composite | M2 EF Category Fluency | Pre-Imputation | 6479 | 620 | 9.57 |
| EF Composite | M2 EF Number Series | Pre-Imputation | 6479 | 624 | 9.63 |
| EF Composite | M2 EF Backward Counting | Pre-Imputation | 6479 | 628 | 9.69 |
| EF Composite | M2 EF SGST | Pre-Imputation | 6479 | 772 | 11.92 |
| EF Composite | M3 EF Digit Backward Scale | Pre-Imputation | 6479 | 1633 | 25.20 |
| EF Composite | M3 EF Category Fluency | Pre-Imputation | 6479 | 1637 | 25.27 |
| EF Composite | M3 EF Number Series | Pre-Imputation | 6479 | 1737 | 26.81 |
| EF Composite | M3 EF Backward Counting | Pre-Imputation | 6479 | 1677 | 25.88 |
| EF Composite | M3 EF SGST | Pre-Imputation | 6479 | 1625 | 25.08 |
| **Domain: Cortisol; Item Type: Cortisol Sample (nmol/L or hr); Missingness Level: Sample-Level (Individual)** | | | | | |
| CAR Mean | Cortisol Waking | Post-Diary-QC Filter | 12958 | 0 | 0 |
| CAR Mean | Cortisol 30 Minutes | Post-Diary-QC Filter | 12958 | 0 | 0 |
| AUC Total Mean | Cortisol At Lunch | Post-Diary-QC Filter | 12958 | 0 | 0 |
| DCS Mean | Cortisol Before Bedtime | Post-Diary-QC Filter | 12958 | 0 | 0 |
| CAR Mean | Cortisol Waking Time | Post-Diary-QC Filter | 12958 | 0 | 0 |
| CAR Mean | Cortisol 30-Minute Time | Post-Diary-QC Filter | 12958 | 0 | 0 |
| AUC Total Mean | Cortisol Lunch Time | Post-Diary-QC Filter | 12958 | 0 | 0 |
| DCS Mean | Cortisol Bedtime | Post-Diary-QC Filter | 12958 | 0 | 0 |

*Note. N* = total sample size; % = percentage of; EF = executive functioning; M2 = Midlife Development in the United States (MIDUS) Wave 2 (2004–2009); SGST = stop-and-go switch task; M3 = MIDUS Wave 3 (2013–2014); CAR = cortisol awakening response; AUC = total area under the curve; DCS = diurnal cortisol slope; QC = quality check.

**Table S3.** ***EF Subtest Wave-Specific Standardization Parameters***

| **Subtest** | **Maximum Range** | **M2 Descriptives** | | **M3 Descriptives** | | **Mean of M3 – M2** | ***SD* Ratio: M3/ M2** |
|---|---|---|---|---|---|---|---|
| | | ***M*** | ***SD*** | ***M*** | ***SD*** | | |
| Digit Backward Span | 8 | 4.946 | 1.385 | 4.864 | 1.343 | -0.082 | 0.969 |
| Category Fluency | 42 | 19.344 | 5.983 | 18.433 | 5.653 | -0.911 | 0.945 |
| Number Series | 5 | 2.377 | 1.486 | 2.191 | 1.476 | -0.186 | 0.993 |
| Backward Counting | 100 | 2.377 | 1.490 | 2.331 | 1.639 | -0.046 | 1.100 |
| Stop-and-Go Switch | 29 | 27.936 | 2.011 | 26.728 | 4.637 | -1.208 | 2.306 |

*Note*. EF = executive functioning; M2 = Midlife Development in the United States (MIDUS) Wave 2 (2004–2009); M3 = MIDUS Wave 3 (2013–2014); *M* = observed mean; *SD* = standard deviation.

**Table S4.** ***EF Composite Descriptive Statistics by Assessment Wave***

| **Wave** | ***N*** | ***N* Persons** | **Mean** | ***SD*** | **Median** | **Minimum** | **Maximum** | **Skewness** |
|---|---|---|---|---|---|---|---|---|
| M2 | 6479 | 1617 | 0.000 | 0.698 | -0.008 | -2.927 | 1.742 | -0.179 |
| M3 | 6479 | 1617 | 0.000 | 0.694 | -0.022 | -2.747 | 2.035 | -0.156 |

*Note*. EF = executive functioning; *N* = sample size; *SD* = standard deviation; M2 = Midlife Development in the United States (MIDUS) Wave 2 (2004–2009); M3 = MIDUS Wave 3 (2013–2014).

**Table S5.** ***Operationalization of Daily Stressor Severity: Construction, Aggregation Logic, and Verification***

| **Dimension** | **Description** |
|---|---|
| MIDUS source variable | B2DSSEVA (Stressor Severity Average) |
| MIDUS dataset / ICPSR study | MIDUS 2 NSDE, ICPSR 26841 |
| Observation unit | Diary day (one row per participant per day; 8 diary days per participant) |
| Number of stressor domains assessed | 7 domains: Argument, Avoided Argument, Work/School, Home, Discrimination, Network, Other |
| Severity items per domain (raw MIDUS names) | Argument, Avoided Argument, Work/School, Home, Discrimination, Network, and Other |
| Severity scale per domain item | 1 (*not at all stressful*) to 4 (*a lot stressful*)–only assessed if that stressor occurred |
| Aggregation within a diary day | Mean of severity ratings across ***all*** stressor domains that occurred on that day (i.e., sum of domain severity ratings/number of stressors occurring) |
| Aggregation across diary days | ***Not*** explicitly averaged across days in the analytic pipeline. The day-level variable is used directly at Level 1 of the LMM. Person-level means were computed post-hoc for descriptive reporting. |
| Value on stressor-free days | 0 (MIDUS assigns 0 when no stressors occurred) |
| Missing-value code and treatment | Code 8 → NA (Refused/Missing) |
| Companion sum variable | sum across occurring domains; missing code 98 → NA |
| Scale range in final data | 0 (stressor-free days) to 4 (maximum stressfulness); confirmed in Part 4 |
| How entered in analytic models | Day-level (Level 1) fixed effect in LMM; also used as person-level moderator in interaction models |
| Standardization | Standardized across pooled person × day dataset |
| Person-level representation in model | Implicit via LMM random intercept (between-person variance captured by random intercept; within-person deviations captured by day-level residual) |

*Note.* MIDUS = Midlife Development in the United States; NSDE = National Survey of Daily Experiences; ICPSR = Inter-university Consortium for Political and Social Research; LMM = linear mixed model; NA = not available.

**Table S6.** ***Individual Stressor Domain Severity Items***

| **Stressor Domain** | ***N* Zero** | **% Zero of Observation** | **Mean (*M*) on Stressor Days** | ***SD* on Stressor Days** |
|---|---|---|---|---|
| Argument / Disagreement | 62 | 4.58 | 2.077 | 0.717 |
| Avoided Argument | 305 | 14.02 | 1.813 | 0.682 |
| Work / School Stress | 102 | 8.46 | 1.995 | 0.637 |
| Home Stress | 70 | 5.69 | 2.112 | 0.660 |
| Discrimination | 17 | 19.54 | 1.929 | 0.748 |
| Network Stress (about someone close) | 27 | 3.55 | 2.082 | 0.628 |
| Other Stress | 82 | 10.12 | 2.019 | 0.665 |

*Note.* *N* = sample size; *M* = observed mean; *SD* = standard deviation.

**Table S7.** ***Daily Stressor Severity by Number of Stressors per Diary Day***

| Day Type | ***N*** **days** | **% of Total** | **Mean (*M*) Severity** | ***SD*** **Severity** | **Median Severity** | **Minimum severity** | **Maximum severity** |
|---|---|---|---|---|---|---|---|
| Stressor-Free Day | 7834 | 60.46 | 1.417 | 0.882 | 1.5 | 0.0 | 3.0 |
| 1 Stressor | 3866 | 29.83 | 1.777 | 0.853 | 2.0 | 0.0 | 3.0 |
| 2 Stressors | 1042 | 8.04 | 1.847 | 0.667 | 2.0 | 0.0 | 3.0 |
| 3+ Stressors | 216 | 1.67 | 1.766 | 0.582 | 1.8 | 0.3 | 3.0 |

*Note.* *N* = sample size; *M* = observed mean; *SD* = standard deviation.

**Table S8.** ***Intraclass Correlation Coefficient (ICC) for Daily Stressor Severity: Variance Decomposition from Unconditional Null Models***

| **Analysis** | ***N* Observations** | ***N* Participants** | **Between-person variance ($\tau_{00}$)** | **Within-person variance ($\sigma^2$)** | **ICC** | **% Between-person** | **% Within-person** | **Interpretation** |
|---|---|---|---|---|---|---|---|---|
| Primary: Wave 0 null model (days nested in participants) | 6479 | 1617 | 0.2042 | 0.5584 | 0.268 | 26.80% | 73.20% | Poor between-person consistency (< .50) |
| Supplementary: All waves pooled (days nested in participants) | 12958 | 1617 | 0.2837 | 0.4788 | 0.372 | 37.20% | 62.80% | Poor (< .50) |
| Test–retest: Wave 0 vs. Wave 1 person-level means (ICC2,1) | 1617 | 1617 | – | – | – | – | – | Not estimable |

*Note.* ICC = intra-class correlation coefficient; *N* = sample size; % = percentage of.

**Table S9.** ***Variance Component Estimates from the Unconditional Null Model for Daily Stressor Severity (Baseline)***

| Component | Value |
|---|---|
| Grand Mean (Fixed Intercept) | 1.56 |
| Between-Person Variance ($\tau_{00}$) | 0.20 |
| Within-Person Variance ($\sigma^2$) | 0.56 |
| Total Variance | 0.76 |
| ICC (Primary, Baseline) | 0.27 |
| % Between-Person | 26.78 |
| % Within-Person | 73.22 |
| *N* Diary Observations | 6479 |
| *N* Participants | 1617 |
| Estimator | REML |

*Note.* ICC = intra-class correlation coefficient; *N* = sample size; REML = restricted maximum likelihood; $\tau_{00}$ = Level-2 (between-person) variance; $\sigma^2$ = Level-1 (within-person/residual) variance. Components extracted from lme4::VarCorr(). Grand mean = fixed intercept from the null model (overall mean stressor severity). Model formula: Stressor ~ 1 + (1 | ID).

**Table S10.** ***Descriptive Statistics for Daily Stressor Severity (Day-Level and Person-Level)***

| Level | *N* Observations | *N* Participants | Mean (*M*) | *SD* | Median | Minimum | Maximum | Skewness | Kurtosis | % Zero |
|---|---|---|---|---|---|---|---|---|---|---|
| Day-level (all observations, wave 0) | 6479 | 1617 | 1.565 | 0.873 | 2.0 | 0.0 | 3.0 | -0.275 | -0.641 | 13.54 |
| Person-level mean (wave 0, mean across diary days) | 1617 | 1617 | 1.565 | 0.586 | 1.5 | 0.0 | 3.0 | -0.061 | -0.188 | 0.49 |

*Note*. *N* = sample size; *M* = observed mean; *SD* = standard deviation; % percentage of; Day-level row = one observation per diary day across all participants; Person-level row = person means computed by averaging across valid diary days; % Zero = percentage of observations where Stressor = 0 (i.e., no stressors endorsed on that diary day). Skewness and kurtosis computed using Fisher’s formula (*psych R* package).

**Table S11.** ***Internal Consistency of the 10-Item GAD Symptom Scale Across MIDUS Waves***

| **Wave** | ***N* Participants** | **Cronbach's α** | **Standardized α** | **α *SE*** | **α LCI** | **α UCI** | **Mean inter-item *r*** | **McDonalds ω total** | **Interpretation** |
|---|---|---|---|---|---|---|---|---|---|
| M2 GAD 10 Items | 4963 | 0.964 | 0.965 | 0.001 | 0.962 | 0.965 | 0.735 | 0.965 | Excellent (≥ .90) |
| M3 GAD 10 Items | 3294 | 0.964 | 0.965 | 0.001 | 0.962 | 0.966 | 0.731 | 0.965 | Excellent (≥ .90) |

*Note*. GAD = generalized anxiety disorder; MIDUS = Midlife Development in the United States; *N* = sample size; α = alpha; LCI = lower bound of the 95% confidence intervals (CIs); UCI = upper bound of the 95% CIs; *r* = correlation coefficient; M2 = Midlife Development in the United States (MIDUS) Wave 2 (2004–2009); M3 = MIDUS Wave 3 (2013–2014).

**Table S12.** ***GAD Item Descriptives at M2 Baseline (Baseline)***

| Item | Item Content | *M* | *(SD)* | Skewness | Kurtosis |
|---|---|---|---|---|---|
| M2 GAD Item 1 | Restless because of worry | 1.338 | 0.724 | 2.291 | 4.637 |
| M2 GAD Item 2 | Keyed up, on edge, or nervous energy | 1.343 | 0.737 | 2.244 | 4.275 |
| M2 GAD Item 3 | Irritable because of worry | 1.352 | 0.745 | 2.232 | 4.242 |
| M2 GAD Item 4 | Trouble falling asleep | 1.350 | 0.800 | 2.329 | 4.330 |
| M2 GAD Item 5 | Trouble staying asleep because of worry | 1.324 | 0.766 | 2.460 | 5.078 |
| M2 GAD Item 6 | Trouble keeping mind on task | 1.322 | 0.727 | 2.434 | 5.258 |
| M2 GAD Item 7 | Trouble remembering things because of worry | 1.254 | 0.647 | 2.822 | 7.645 |
| M2 GAD Item 8 | Low on energy | 1.349 | 0.800 | 2.332 | 4.345 |
| M2 GAD Item 9 | Tired easily because of worry | 1.305 | 0.749 | 2.563 | 5.629 |
| M2 GAD Item 10 | Sore or aching muscles because of tension | 1.279 | 0.726 | 2.740 | 6.589 |

*Note*. GAD = generalized anxiety disorder; M2 = Midlife Development in the United States (MIDUS) Wave 2 (2004–2009); *M* = observed mean; *SD* = standard deviation.

**Table S13.** ***Test–Retest Reliability of GAD Severity: M2 to M3 (9-Year Interval)***

| **Statistic** | **Value** |
|---|---|
| *N* participants (both waves non-missing) | 3294 |
| M2 GAD Severity – *M (SD)* | 0.127 (0.866) |
| M3 GAD Severity – *M (SD)* | 0.131 (0.921) |
| Pearson *r* | 0.424 |
| Pearson *r* 95% CI lower | 0.395 |
| Pearson *r* 95% CI upper | 0.451 |
| Pearson *r* *p*-value | .000 |
| $ICC_{2,1}$ (two-way mixed, absolute, single) | 0.423 |
| $ICC_{2,1}$ 95% CI lower | 0.395 |
| $ICC_{2,1}$ 95% CI upper | 0.451 |
| $ICC_{2,1}$ *p*-value | .000 |
| $ICC_{3,1}$ (two-way mixed, consistency, single) | 0.423 |
| $ICC_{3,1}$ 95% CI lower | 0.395 |
| $ICC_{3,1}$ 95% CI upper | 0.451 |
| Mean change (M3 − M2) | 0.005 |
| *SD* of change | 0.960 |
| Paired *t*-test *p*-value | .786 |
| Stability interpretation (9-year interval) | Moderate stability (.30–.49) |

*Note.* GAD = generalized anxiety disorder; M2 = Midlife Development in the United States (MIDUS) Wave 2 (2004–2009); M3 = MIDUS Wave 3 (2013–2014); *M* = observed mean; *SD* = standard deviation; CI = 95% confidence intervals; ICC = intra-class correlation coefficients. Composite scores are MIDUS pre-computed summed/averaged ratings from the 10 worry-symptom items at each wave. Test–retest interval ≈ 9 years (M2 to M3). Pearson *r* = test–retest correlation with 95% CI. $ICC_{2,1}$ = two-way mixed, absolute agreement, single measures (Shrout & Fleiss, 1979). $ICC_{3,1}$ = two-way mixed, consistency, single measures. A significant paired *t*-test would indicate systematic mean-level change across waves.

**Table S14.** ***Variance Component Estimates from the Unconditional Null Model for the EF Composite***

| **Component** | **Value** |
|---|---|
| Model | Null |
| Outcome | EF composite (mean of 5 *z*-scored subtests) |
| Grouping factor | Participant ID |
| Estimator | REML |
| *N* Observations | 12958 |
| *N* Participants | 1617 |
| Grand mean (fixed intercept) | 0.000 |
| Between-person variance ($\tau_{00}$) | 0.391 |
| Within-person variance ($\sigma^2$) | 0.093 |
| Total variance | 0.484 |
| ICC (unconditional) | 0.808 |
| % Between-person | 80.81 |
| % Within-person | 19.19 |
| Interpretation | Large between-person clustering (≥ .50) |

*Note.* EF = executive functioning; ID = unique identifier; REML = restricted maximum likelihood; *N* = sample size; ICC = intra-class correlation coefficient; % = percentage of.

**Table S15.** ***Within-Model Conditional ICCs Across Models 1–5***

| **Model** | **ICC (within model, conditional)** | **Var. component 1 ($\tau_{00}$ residual)** | **Sum of all var. components** | ***N* fixed effects in model** |
|---|---|---|---|---|
| Model 1: Time + Covariates | 0.771 | 0.394 | 0.511 | 12 |
| Model 2: Main Effects + Time | 0.771 | 0.391 | 0.507 | 16 |
| Model 3: CAR × Stressor × Time (Primary) | 0.771 | 0.391 | 0.507 | 18 |
| Model 4: Random Slopes (Stressor) | 0.812 | 0.374 | 0.461 | 18 |
| Model 5: Random Slopes (CAR) | 0.752 | 0.390 | 0.518 | 18 |

*Note*. ICC = intra-class correlation coefficient; Var. = variance; *N* = sample size; CAR = cortisol awakening response.

**Table S16.** ***Model Comparison Statistics for LMMs and GAMMs Examining Cortisol Dysregulation Indices as Moderators of the Association Between Daily Stressor Severity and 9-Year Executive Functioning***

| Model | Type | AIC | BIC | Log-Lik | Deviance | $R^2$ (Marginal) | $R^2$ (Conditional) | ICC | Deviance explained | ΔAIC | ΔBIC |
|---|---|---|---|---|---|---|---|---|---|---|---|
| **A. AUC × Daily Stress Severity** | | | | | | | | | | | |
| M4: Random Slopes (Stressor) | LMM | -1377.4 | -1190.7 | 713.7 | -1427.4 | 26.6% | 97.0% | 77.2% | – | 0.0 | 0.0 |
| M1: Time + Covariates | LMM | -686.1 | -566.6 | 359.1 | -718.1 | 26.2% | 96.2% | 77.1% | – | 691.3 | 624.1 |
| M2: Main Effects + Time | LMM | -672.4 | -523.0 | 356.2 | -712.4 | 26.1% | 96.2% | 77.1% | – | 705.1 | 667.7 |
| M5: Random Slopes (AUC) | LMM | -655.5 | -468.8 | 352.8 | -705.5 | 25.9% | 96.2% | 75.2% | – | 721.9 | 721.9 |
| M3: AUC × Stressor × Time (Primary) | LMM | -653.5 | -489.2 | 348.8 | -697.5 | 26.1% | 96.2% | 77.1% | – | 723.9 | 701.5 |
| M7: GAMM te Interaction | GAMM | 6875.9 | 18582.8 | -1870.7 | 1012.6 | – | 81.6% | – | 83.8 | 8253.4 | 19773.5 |
| M6: GAMM ti Interaction | GAMM | 6876.9 | 18581.6 | -1871.5 | 1012.8 | – | 81.6% | – | 83.8 | 8254.3 | 19772.3 |
| **B. CAR × Daily Stress Severity** | | | | | | | | | | | |
| M4: Random Slopes (Stressor) | LMM | -1374.8 | -1188.0 | 712.4 | -1424.8 | 26.6% | 97.0% | 77.2% | – | 0.0 | 0.0 |
| M1: Time + Covariates | LMM | -686.1 | -566.6 | 359.1 | -718.1 | 26.2% | 96.2% | 77.1% | – | 688.7 | 621.4 |
| M2: Main Effects + Time | LMM | -670.4 | -521.0 | 355.2 | -710.4 | 26.2% | 96.2% | 77.1% | – | 704.4 | 667.0 |
| M5: Random Slopes (CAR) | LMM | -648.6 | -461.9 | 349.3 | -698.6 | 26.1% | 96.2% | 75.7% | – | 726.2 | 726.2 |
| M3: CAR × Stressor × Time (Primary) | LMM | -647.4 | -483.1 | 345.7 | -691.4 | 26.2% | 96.2% | 77.1% | – | 727.4 | 704.9 |
| M7: GAMM te Interaction | GAMM | 6877.4 | 18584.2 | -1871.4 | 1012.8 | – | 81.6% | – | 83.8 | 8252.2 | 19772.2 |
| M6: GAMM ti Interaction | GAMM | 6881.8 | 18602.1 | -1871.8 | 1012.8 | – | 81.6% | – | 83.8 | 8256.5 | 19790.1 |
| **C. DCS × Daily Stress Severity** | | | | | | | | | | | |
| M4: Random Slopes (Stressor) | LMM | -1386.0 | -1199.2 | 718.0 | -1436.0 | 27.3% | 97.0% | 77.0% | – | 0.0 | 0.0 |
| M1: Time + Covariates | LMM | -686.1 | -566.6 | 359.1 | -718.1 | 26.2% | 96.2% | 77.1% | – | 699.9 | 632.6 |
| M2: Main Effects + Time | LMM | -677.8 | -528.4 | 358.9 | -717.8 | 26.8% | 96.2% | 77.0% | – | 708.2 | 670.9 |
| M3: DCS × Stressor × Time (Primary) | LMM | -667.9 | -503.6 | 356.0 | -711.9 | 26.8% | 96.2% | 77.0% | – | 718.0 | 695.6 |
| M5: Random Slopes (DCS) | LMM | -663.0 | -476.2 | 356.5 | -713.0 | 87.6% | – | 76.1% | – | 723.0 | 723.0 |
| M6: GAMM ti Interaction | GAMM | 6875.4 | 18579.2 | -1870.8 | 1012.7 | – | 81.6% | – | 83.8 | 8261.3 | 19778.4 |
| M7: GAMM te Interaction | GAMM | 6875.5 | 18579.7 | -1870.8 | 1012.7 | – | 81.6% | – | 83.8 | 8261.4 | 19778.9 |

Note. AIC = Akaike Information Criterion; BIC = Bayesian Information Criterion; Log-Lik = log-likelihood; ICC = Intraclass Correlation Coefficient; ΔAIC = change in AIC relative to the best-fitting model; ΔBIC = change in BIC relative to the best-fitting model. LMM = Linear Mixed Model estimated via restricted maximum likelihood (REML). GAMM = Generalized Additive Mixed Model. te = tensor product smooth interaction (full interaction between predictors); ti = tensor product interaction smooth (interaction component only, with main effects estimated separately). $R^2$ (Marginal) = variance explained by fixed effects only; $R^2$ (Conditional) = variance explained by both fixed and random effects (Nakagawa, Schielzeth, & O'Hara, 2012). ICC reflects the proportion of total variance attributable to between-person differences. These indices are not reported for GAMMs, for which deviance explained is provided instead. AUC = total area under the cortisol curve with respect to ground, reflecting overall cortisol output; CAR = cortisol awakening response, reflecting the rise in cortisol in the first 30–45 minutes after waking; DCS = diurnal cortisol slope, reflecting the rate of cortisol decline across the day. Models are ordered within each panel by Akaike Information Criterion (AIC); lower AIC values indicate better fit. ΔAIC and ΔBIC represent the difference from the best-fitting model within each panel. The best-fitting model (M4: Random Slopes [Stressor]) is shown first in each panel.

**Table S17.** ***Model Comparison Statistics for LMMs and GAMMs Examining Cortisol Dysregulation Indices as Moderators of the Association Between Chronic Worry Severity and 9-Year Executive Functioning***

| Model | Type | AIC | BIC | Log-Lik | Deviance | $R^2$ (Marginal) | $R^2$ (Conditional) | ICC | Deviance explained | ΔAIC | ΔBIC |
|---|---|---|---|---|---|---|---|---|---|---|---|
| **A. AUC × Chronic Worry Severity** | | | | | | | | | | | |
| M4: Random Slopes (Worry) | LMM | -1192.1 | -1005.4 | 621.1 | -1242.1 | 25.1% | 96.4% | 81.2% | – | 0.0 | 0.0 |
| M1: Time + Covariates | LMM | -762.9 | -613.5 | 401.5 | -802.9 | 25.7% | 96.2% | 77.1% | – | 429.2 | 391.9 |
| M2: Main Effects + Time | LMM | -751.7 | -564.9 | 400.8 | -801.7 | 86.9% | – | 75.2% | – | 440.5 | 440.5 |
| M5: Random Slopes (AUC) | LMM | -749.8 | -585.5 | 396.9 | -793.8 | 25.7% | 96.2% | 77.1% | – | 442.3 | 419.9 |
| M3: AUC × Worry × Time (Primary) | LMM | -686.1 | -566.6 | 359.1 | -718.1 | 26.2% | 96.2% | 77.1% | – | 506.0 | 438.8 |
| M7: GAMM te Interaction | GAMM | 6834.9 | 18559.3 | -1847.8 | 1009.1 | – | 81.7% | – | 83.9 | 8027.0 | 19564.7 |
| M6: GAMM ti Interaction | GAMM | 6835.2 | 18552.5 | -1848.9 | 1009.3 | – | 81.7% | – | 83.9 | 8027.3 | 19557.9 |
| **B. CAR× Chronic Worry Severity** | | | | | | | | | | | |
| M4: Random Slopes (Worry) | LMM | -1188.3 | -1001.6 | 619.2 | -1238.3 | 25.3% | 96.5% | 81.7% | – | 0.0 | 0.0 |
| M1: Time + Covariates | LMM | -763.1 | -598.8 | 403.5 | -807.1 | 25.7% | 96.2% | 77.1% | – | 425.2 | 402.8 |
| M2: Main Effects + Time | LMM | -763.0 | -576.2 | 406.5 | -813.0 | 25.6% | 96.2% | 76.0% | – | 425.4 | 425.4 |
| M5: Random Slopes (CAR) | LMM | -761.2 | -611.8 | 400.6 | -801.2 | 25.8% | 96.2% | 77.1% | – | 427.2 | 389.8 |
| M3: CAR × Worry × Time (Primary) | LMM | -686.1 | -566.6 | 359.1 | -718.1 | 26.2% | 96.2% | 77.1% | – | 502.2 | 435.0 |
| M7: GAMM te Interaction | GAMM | 6825.1 | 18541.7 | -1844.0 | 1008.5 | – | 81.7% | – | 83.9 | 8013.4 | 19543.3 |
| M6: GAMM ti Interaction | GAMM | 6825.9 | 18546.5 | -1843.8 | 1008.5 | – | 81.7% | – | 83.9 | 8014.3 | 19548.1 |
| **C. DCS × Chronic Worry Severity** | | | | | | | | | | | |
| M4: Random Slopes (Worry) | LMM | -1193.4 | -1006.6 | 621.7 | -1243.4 | 25.5% | 96.5% | 81.0% | – | 0.0 | 0.0 |
| M1: Time + Covariates | LMM | -769.5 | -620.1 | 404.7 | -809.5 | 26.5% | 96.2% | 77.0% | – | 423.9 | 386.6 |
| M2: Main Effects + Time | LMM | -765.0 | -600.7 | 404.5 | -809.0 | 26.5% | 96.2% | 77.0% | – | 428.3 | 405.9 |
| M3: DCS × Worry × Time (Primary) | LMM | -760.0 | -573.2 | 405.0 | -810.0 | 87.4% | – | 76.2% | – | 433.4 | 433.4 |
| M5: Random Slopes (DCS) | LMM | -686.1 | -566.6 | 359.1 | -718.1 | 26.2% | 96.2% | 77.1% | – | 507.2 | 440.0 |
| M6: GAMM ti Interaction | GAMM | 6828.3 | 18551.0 | -1844.7 | 1008.6 | – | 81.7% | – | 83.9 | 8021.6 | 19557.6 |
| M7: GAMM te Interaction | GAMM | 6831.4 | 18544.5 | -1847.6 | 1009.0 | – | 81.7% | – | 83.9 | 8024.8 | 19551.1 |

*Note*. AIC = Akaike Information Criterion; BIC = Bayesian Information Criterion; Log-Lik = log-likelihood; ICC = Intraclass Correlation Coefficient; ΔAIC = change in AIC relative to the best-fitting model; ΔBIC = change in BIC relative to the best-fitting model. LMM = Linear Mixed Model estimated via restricted maximum likelihood (REML); GAMM = Generalized Additive Mixed Model. te = tensor product smooth interaction (full interaction between predictors); ti = tensor product interaction smooth (interaction component only, with main effects estimated separately). $R^2$ (Marginal) = variance explained by fixed effects only; $R^2$ (Conditional) = variance explained by both fixed and random effects (Nakagawa et al., 2012). ICC reflects the proportion of total variance attributable to between-person differences. These indices are not reported for GAMMs, for which deviance explained is provided instead. AUC = total area under the cortisol curve with respect to ground, reflecting overall cortisol output; CAR = cortisol awakening response, reflecting the rise in cortisol in the first 30–45 minutes after waking; DCS = diurnal cortisol slope, reflecting the rate of cortisol decline across the day. Models are ordered within each panel by Akaike Information Criterion (AIC); lower AIC values indicate better fit. ΔAIC and ΔBIC represent the difference from the best-fitting model within each panel. The best-fitting model (M4: Random Slopes [Worry]) is shown first in each panel.

**Table S18.** ***Full Fixed-Effect Estimates: Primary vs. Sensitivity Model***

| **Term** | ***b*** | ***SE*** | ***t*** | ***df*** | ***p*** |
|---|---|---|---|---|---|
| **Model 3 Primary (all covariates)** | | | | | |
| Intercept | 0.006 | 0.023 | 0.257 | 1853 | .797 |
| Time | 0.000 | 0.012 | -0.030 | 1615 | .976 |
| z(Slope Mean) | 0.006 | 0.016 | 0.361 | 1605 | .718 |
| z(Stressor Severity) | 0.010 | 0.002 | 4.081 | 10018 | .000 |
| z(Age) | -0.203 | 0.014 | -14.398 | 1633 | .000 |
| Women vs. Men | -0.057 | 0.028 | -2.012 | 1608 | .044 |
| z(Global Cognition) | 0.004 | 0.014 | 0.283 | 1588 | .777 |
| z(Self-Rated Health) | 0.007 | 0.003 | 2.049 | 10413 | .041 |
| z(Chronic Illnesses) | -0.104 | 0.008 | -12.896 | 11076 | .000 |
| z(Prescription Medications) | 0.004 | 0.006 | 0.658 | 11125 | .511 |
| z(Non-Prescription Medications) | -0.139 | 0.009 | -15.587 | 11299 | .000 |
| Sleep disturbances | -0.024 | 0.004 | -6.351 | 10486 | .000 |
| Smoking status | 0.053 | 0.007 | 8.041 | 10471 | .000 |
| z(Body Mass Index) | -0.063 | 0.005 | -13.922 | 11036 | .000 |
| z(Slope Mean) × z(Stressor Severity) | 0.000 | 0.002 | -0.151 | 9984 | .880 |
| Time × z(Slope Mean) | -0.007 | 0.012 | -0.533 | 1615 | .594 |
| Time × z(Stressor Severity)) | -0.002 | 0.003 | -0.646 | 10275 | .519 |
| Time × z(Slope Mean) × z(Stressor Severity) | -0.003 | 0.003 | -0.785 | 10199 | .432 |
| **Model 3 Sensitivity (no sleep/smoking/BMI)** | | | | | |
| Intercept | 0.024 | 0.023 | 1.040 | 1787 | .298 |
| Time | 0.000 | 0.012 | -0.030 | 1615 | .976 |
| z(Slope Mean) | 0.003 | 0.016 | 0.173 | 1604 | .862 |
| z(Stressor Severity) | 0.009 | 0.002 | 3.571 | 10032 | .000 |
| z(Age) | -0.200 | 0.014 | -13.888 | 1629 | .000 |
| Women vs. Men | -0.042 | 0.029 | -1.465 | 1605 | .143 |
| z(Global Cognition) | 0.007 | 0.014 | 0.492 | 1589 | .623 |
| z(Self-Rated Health) | 0.003 | 0.003 | 0.758 | 10400 | .448 |
| z(Chronic Illnesses) | -0.134 | 0.008 | -16.954 | 11089 | .000 |
| z(Prescription Medications) | 0.004 | 0.006 | 0.605 | 11112 | .545 |
| z(Non-Prescription Medications) | -0.168 | 0.009 | -19.057 | 11300 | .000 |
| z(Slope Mean) × z(Stressor Severity) | -0.001 | 0.002 | -0.194 | 9995 | .846 |
| Time × z(Slope Mean) | -0.007 | 0.012 | -0.534 | 1615 | .594 |
| Time × z(Stressor Severity)) | -0.002 | 0.003 | -0.636 | 10293 | .525 |
| Time × z(Slope Mean) × z(Stressor Severity) | -0.003 | 0.003 | -0.773 | 10215 | .439 |

*Note.* DCS = diurnal cortisol slope; LMM = linear mixed modeling; *b* = unstandardized regression estimate; *SE* = standard error; *t* = *t*-statistic; *df* = degrees of freedom.

**Table S19.** ***Model Fit: Primary vs. Sensitivity Model***

| **Model** | ***N* Fixed Effects** | **AIC** | **BIC** | **LL** | ***R²* Marginal** | ***R²* Conditional** | **ΔAIC** | **ΔBIC** |
|---|---|---|---|---|---|---|---|---|
| Model 3 Primary (Groups A + B) | 18 | -647.4 | -483.1 | 345.7 | 26.20% | 96.20% | 0.0 | 0.0 |
| Model 3 Sensitivity (Group A) | 15 | -367.7 | -225.8 | 202.8 | 25.70% | 96.20% | 279.7 | 257.3 |

*Note*. *N* = sample size; AIC = Akaike information criterion; BIC = Bayesian information criterion; LL = log-likelihood; $R^2$ = R-squared; Δ = change in statistic.

**Table S20.** ***Raw-Score Probe Values and Sample Distribution of Moderator Variables at Low (−1 SD), Mean (0 SD), and High (+1 SD) Probing Levels***

| **Cortisol metric** | **Moderator** | **Probe level** | **Raw score** | ***N* in region** | **% in region** | **Region definition** |
|---|---|---|---|---|---|---|
| CAR | Daily Stressor Severity | Low (-1 *SD*) | 0.721 | 225 | 13.9 | Persons scoring ≤ 0.721 (at or below -1 *SD*) |
| CAR | Daily Stressor Severity | Mean (0 *SD*) | 1.584 | 1162 | 71.9 | Persons scoring > 0.721 and < 2.448 (-1 *SD* to +1 *SD*) |
| CAR | Daily Stressor Severity | High (+1 *SD*) | 2.448 | 230 | 14.2 | Persons scoring ≥ 2.448 (at or above +1 *SD*) |
| DCS | Chronic Worry / GAD Symptom Severity | Low (-1 *SD*) | -0.736 | 0 | 0 | Persons scoring ≤ -0.736 (at or below -1 *SD*) |
| DCS | Chronic Worry / GAD Symptom Severity | Mean (0 *SD*) | 0.142 | 1570 | 97.1 | Persons scoring > -0.736 and < 1.019 (-1 *SD* to +1 *SD*) |
| DCS | Chronic Worry / GAD Symptom Severity | High (+1 *SD*) | 1.019 | 47 | 2.9 | Persons scoring ≥ 1.019 (at or above +1 *SD*) |
| AUC | Chronic Worry / GAD Symptom Severity | Low (-1 *SD*) | -0.736 | 0 | 0 | Persons scoring ≤ -0.736 (at or below -1 *SD*) |
| AUC | Chronic Worry / GAD Symptom Severity | Mean (0 *SD*) | 0.142 | 1570 | 97.1 | Persons scoring > -0.736 and < 1.019 (-1 *SD* to +1 *SD*) |
| AUC | Chronic Worry / GAD Symptom Severity | High (+1 *SD*) | 1.019 | 47 | 2.9 | Persons scoring ≥ 1.019 (at or above +1 *SD*) |

*Note*. *SD* = standard deviation; *N* = sample size; CAR = cortisol awakening response; DCS = diurnal cortisol slope; GAD = generalized anxiety disorder; AUC = total cortisol area under the curve; MIDUS = Midlife Development in the United States. Probe levels are exact raw-score values at which the interaction is evaluated, not distributional subgroups or quantile cut-points. Raw score = mean ± 1×*SD* of the person-level moderator distribution. N in region and % in region report how many participants fall at or below the Low probe, between Low and High probes, or at or above the High probe. Daily Stressor Severity: daily diary mean perceived stressor severity, scale 0–3. Chronic Worry / GAD Symptom Severity: MIDUS 2 GAD composite (mean of 10 items). Blue rows = Low (−1 *SD*) probe. Grey = Mean (0 *SD*). Pink = High (+1 *SD*).

**Table S21.** ***Random Effects Variance Components from the Best-Fitting LMMs Examining Cortisol Dysregulation Indices as Moderators of the Within-Person Association Between Daily Stressor Severity or Chronic Worry Severity and 9-Year Executive Functioning***

| Group | Term | $\tau^2$ | Variance | *SD* | ICC |
|---|---|---|---|---|---|
| **A. AUC × Daily Stress Severity** | | | | | |
| Participant ID | Intercept | – | 0.394 | 0.628 | 77.1% |
| Participant ID | Time | – | 0.240 | 0.490 | 47.0% |
| Participant ID | Intercept | Time | -0.143 | -0.466 | -28.1% |
| Residual | – | – | 0.020 | 0.142 | 4.0% |
| **B. CAR × Daily Stress Severity** | | | | | |
| Participant ID | Intercept | – | 0.394 | 0.628 | 77.1% |
| Participant ID | Time | – | 0.240 | 0.490 | 47.0% |
| Participant ID | Intercept | Time | -0.144 | -0.467 | -28.1% |
| Residual | – | – | 0.020 | 0.142 | 4.0% |
| **C. DCS × Daily Stress Severity** | | | | | |
| Participant ID | Intercept | – | 0.392 | 0.626 | 77.0% |
| Participant ID | Time | – | 0.240 | 0.490 | 47.1% |
| Participant ID | Intercept | Time | -0.143 | -0.466 | -28.1% |
| Residual | – | – | 0.020 | 0.142 | 4.0% |
| **D. AUC × Chronic Worry Severity** | | | | | |
| Participant ID | Intercept | – | 0.391 | 0.625 | 77.1% |
| Participant ID | Time | – | 0.241 | 0.490 | 47.5% |
| Participant ID | Intercept | Time | -0.145 | -0.471 | -28.5% |
| Residual | – | – | 0.020 | 0.142 | 3.9% |
| **E. CAR × Chronic Worry Severity** | | | | | |
| Participant ID | Intercept | – | 0.391 | 0.625 | 77.1% |
| Participant ID | Time | – | 0.240 | 0.490 | 47.4% |
| Participant ID | Intercept | Time | -0.144 | -0.470 | -28.4% |
| Residual | – | – | 0.020 | 0.141 | 3.9% |
| **F. DCS × Chronic Worry Severity** | | | | | |
| Participant ID | Intercept | – | 0.389 | 0.624 | 77.0% |
| Participant ID | Time | – | 0.241 | 0.490 | 47.6% |
| Participant ID | Intercept | Time | -0.144 | -0.472 | -28.6% |
| Residual | – | – | 0.020 | 0.142 | 4.0% |

*Note*. AUC = total area under the cortisol curve with respect to ground; CAR = cortisol awakening response; DCS = diurnal cortisol slope; ICC = intraclass correlation coefficient, expressing each variance component as a percentage of total variance; REML = restricted maximum likelihood; *SD* = standard deviation of the random effect (square root of the variance, or for the covariance term, the signed square root); $\tau^2$ = covariance between random effects (i.e., the intercept–slope covariance); Variance = estimated variance component for each random effect. "–" indicates the term is a variance rather than a covariance. Random effects are from the best-fitting model (M4: Random Slopes [Stressor] for the daily stressor severity panels and Random Slopes [Worry] for the chronic worry panels; see Tables S16–S17) for each cortisol metric. All models included random intercepts and random slopes for daily stressor severity (Time), estimated using REML. Participant ID rows reflect between-person variability; the Residual row reflects within-person variability not accounted for by the model. Negative ICC values for the Intercept–Time covariance reflect a negative correlation between individual intercepts and slopes (i.e., participants with higher baseline executive functioning tended to show steeper declines across the 9-year interval). The sum of ICC values does not total 100% because the negative intercept–slope covariance partially offsets the variance components.

**Table S22.** ***Empirical Verification: Binary Time (0/1) vs. Elapsed-Year (~0/9) Model Comparison–Fixed Effects***

| **Time Definition** | **Binary** | | | | **Years** | | | | **Expected** | **Observed** |
|---|---|---|---|---|---|---|---|---|---|---|
| **Term** | ***b*** | ***SE*** | ***t*** | ***p*** | ***b*** | ***SE*** | ***t*** | ***p*** | **ratio** | **ratio** |
| Intercept | 0.003 | 0.022 | 0.137 | .891 | 0.006 | 0.023 | 0.257 | .797 | 1 (unchanged) | 1.896 |
| Time | -0.001 | 0.012 | -0.055 | .956 | 0.000 | 0.001 | -0.030 | .976 | 1/9 | 0.061 |
| *z*(Stressor Severity) | -0.039 | 0.006 | -6.089 | .000 | 0.010 | 0.002 | 4.081 | .000 | 1 (unchanged) | -0.250 |
| *z*(Age) | -0.213 | 0.014 | -15.370 | .000 | -0.203 | 0.014 | -14.398 | .000 | 1 (unchanged) | 0.953 |
| Women vs. Men | -0.050 | 0.028 | -1.788 | .074 | -0.057 | 0.028 | -2.012 | .044 | 1 (unchanged) | 1.136 |
| *z*(Global Cognition) | 0.004 | 0.014 | 0.302 | .762 | 0.004 | 0.014 | 0.283 | .777 | 1 (unchanged) | 0.946 |
| *z*(Self-Rated Health) | 0.012 | 0.003 | 3.581 | .000 | 0.007 | 0.003 | 2.049 | .040 | 1 (unchanged) | 0.567 |
| *z*(Chronic Illnesses) | -0.092 | 0.008 | -11.320 | .000 | -0.104 | 0.008 | -12.896 | .000 | 1 (unchanged) | 1.130 |
| *z*(Prescription Medications) | -0.005 | 0.006 | -0.735 | .462 | 0.004 | 0.006 | 0.658 | .510 | 1 (unchanged) | -0.891 |
| *z*(Non-Prescription Medications) | -0.126 | 0.009 | -14.103 | .000 | -0.139 | 0.009 | -15.587 | .000 | 1 (unchanged) | 1.101 |
| Sleep disturbances | -0.019 | 0.004 | -4.980 | .000 | -0.024 | 0.004 | -6.351 | .000 | 1 (unchanged) | 1.283 |
| Smoking status | 0.054 | 0.007 | 8.134 | .000 | 0.053 | 0.007 | 8.041 | .000 | 1 (unchanged) | 0.993 |
| *z*(Body Mass Index) | -0.053 | 0.005 | -11.440 | .000 | -0.063 | 0.005 | -13.922 | .000 | 1 (unchanged) | 1.189 |
| Time × z(Stressor Severity) | 0.020 | 0.008 | 2.656 | .008 | 0.000 | 0.000 | -0.646 | .519 | 1/9 | -0.012 |

*Note*. *b* = unstandardized regression estimate; *SE* = standard error; *t* = *t*-statistic; *p* = probability value; *z* = standardized parameter estimates.

**Table S23.** ***Full Specification of Model 3 (CAR Example): Cortisol Awakening Response by Chronic Worry Severity by Time Interaction, Testing Hypothesis 2***

| Model | Equation |
|---|---|
| Level-1 equation | EF_ij (Executive Functioning) = $\pi_{0j}$ (Intercept) + $\pi_{1j}$ (Time Slope) × TIME_ij (Time) + e_ij (Residual) |
| Level-2: $\pi_{0j}$ (Intercept) | $\pi_{0j}$ (Intercept) = $\gamma_{00}$ (Grand Intercept) + $\gamma_{20}$ (CAR Main Effect) × CAR_j (Cortisol Awakening Response) + $\gamma_{40}$ (Worry Main Effect) × WORRY_j (Chronic Worry Severity) + $\gamma_{60}$ (CAR × Worry Interaction) × CAR_j (Cortisol Awakening Response) × WORRY_j (Chronic Worry Severity) + $\Sigma_k(\gamma_{k0}$ (Covariate Effect) × X_kj (Covariate)) + $u_{0j}$ (Random Intercept) |
| Level-2: $\pi_{1j}$ (Time Slope) | $\pi_{1j}$ (Time Slope) = $\gamma_{10}$ (Time Main Effect) + $\gamma_{30}$ (CAR × Time Interaction) × CAR_j (Cortisol Awakening Response) + $\gamma_{50}$ (Worry × Time Interaction) × WORRY_j (Chronic Worry Severity) + $\gamma_{70}$ (CAR × Worry × Time Interaction) × CAR_j (Cortisol Awakening Response) × WORRY_j (Chronic Worry Severity) + $u_{1j}$ (Random Time Slope) [← PRIMARY TEST, HYPOTHESIS 2] |
| Combined form (Eq. 3) | EF_ij (Executive Functioning) = $\gamma_{00}$ (Grand Intercept) + $\gamma_{10}$ (Time Main Effect) × TIME (Time)<br><br>• $\gamma_{20}$ (CAR Main Effect) × CAR (Cortisol Awakening Response) + $\gamma_{30}$ (CAR × Time Interaction) × CAR (Cortisol Awakening Response) × TIME (Time)<br>• $\gamma_{40}$ (Worry Main Effect) × WORRY (Chronic Worry Severity) + $\gamma_{50}$ (Worry × Time Interaction) × WORRY (Chronic Worry Severity) × TIME (Time)<br>• $\gamma_{60}$ (CAR × Worry Interaction) × CAR (Cortisol Awakening Response) × WORRY (Chronic Worry Severity)<br>• $\gamma_{70}$ (CAR × Worry × Time Interaction) × CAR (Cortisol Awakening Response) × WORRY (Chronic Worry Severity) × TIME (Time) [3-WAY PRIMARY, HYPOTHESIS 2]<br>• $\Sigma_k(\gamma_{k0}$ (Covariate Effect) × X_k (Covariate)) + $u_{0j}$ (Random Intercept) + $u_{1j}$ (Random Time Slope) × TIME (Time) + e_ij (Residual) |
| Primary parameter | $\gamma_{70}$ (CAR × Worry × Time Interaction): three-way CAR (Cortisol Awakening Response) × WORRY (Chronic Worry Severity) × TIME interaction. Whether chronic worry severity moderates the CAR–EF-change association over nine years. Negative $\gamma_{70}$ ⇒ blunted CAR (Cortisol Awakening Response) combined with high WORRY (Chronic Worry Severity) ⇒ steeper EF decline. |
| Random effects | ($u_{0j}$ (Random Intercept), $u_{1j}$ (Random Time Slope)) ~ N(0, $\Sigma_u$ 2×2 unstructured); e_ij (Residual) ~ N(0, $\sigma^2_e$) |

*Note.* CAR = cortisol awakening response.

**Table S24.** ***Formal Difference Tests Between Low (-1 SD) and High (+1 SD) Simple Slopes of Cortisol Dysregulation on 9-Year Executive Functioning, Across All Six Cortisol Metric x Moderator Models***

| Low simple slope (*b*) | Low *SE* | High simple slope (*b*) | High *SE* | Covariance (Low, High) | Correlation (Low, High) | Difference (High − Low) | *SE* of difference | *z* | *p* | LCI | UCI |
|---|---|---|---|---|---|---|---|---|---|---|---|
| **CAR x Daily Stressor Severity (Low vs. High)** | | | | | | | | | | | |
| 0.002 | 0.015 | -0.004 | 0.015 | 0.0002 | 0.952 | -0.006 | 0.005 | -1.256 | 0.209 | -0.015 | 0.003 |
| **DCS x Daily Stressor Severity (Low vs. High)** | | | | | | | | | | | |
| -0.038 | 0.015 | -0.026 | 0.015 | 0.0002 | 0.951 | 0.012 | 0.005 | 2.496 | 0.013 | 0.003 | 0.021 |
| **AUC x Daily Stressor Severity (Low vs. High)** | | | | | | | | | | | |
| -0.011 | 0.015 | -0.020 | 0.015 | 0.0002 | 0.953 | -0.009 | 0.005 | -1.928 | 0.054 | -0.018 | 0.000 |
| **CAR x Chronic Worry Severity (Low vs. High)** | | | | | | | | | | | |
| 0.023 | 0.016 | -0.027 | 0.017 | 0.0002 | 0.601 | -0.050 | 0.015 | -3.350 | 0.001 | -0.079 | -0.021 |
| **DCS x Chronic Worry Severity (Low vs. High)** | | | | | | | | | | | |
| -0.022 | 0.016 | -0.046 | 0.015 | 0.0002 | 0.834 | -0.023 | 0.009 | -2.631 | 0.009 | -0.041 | -0.006 |
| **AUC x Chronic Worry Severity (Low vs. High)** | | | | | | | | | | | |
| -0.017 | 0.016 | -0.012 | 0.016 | 0.0002 | 0.812 | 0.004 | 0.010 | 0.442 | 0.659 | -0.015 | 0.023 |

*Note*. *SE* = standard error; *z* = Wald test statistic; *p* = two-tailed *p*-value; 95% CI = confidence interval for the difference; CAR = cortisol awakening response; AUC = total area under the curve; DCS = diurnal cortisol slope. Difference = High simple slope minus Low simple slope, computed as a linear contrast on each model's fixed effects using the full variance-covariance matrix (delta method), correctly accounting for the non-independence of the two simple slopes within each model. Cov(Low, High) and Corr(Low, High) are reported for transparency regarding non-independence. In the three Chronic Worry Severity models, the moderator term 'Daily Stressor Severity' refers to z-scored chronic worry/GAD severity, not daily stressor severity; this matches the underlying variable construction used throughout the primary analysis. These tests are distinct from individual-slope significance (Table 3) and from the overlapping-CI heuristic reported in the manuscript text.

References

Nakagawa, S., Schielzeth, H., & O'Hara, R. B. (2012). A general and simple method for obtaining R2 from generalized linear mixed-effects models. *Methods in Ecology and Evolution, 4*, 133-142. doi:10.1111/j.2041-210x.2012.00261.x

Shrout, P. E., & Fleiss, J. L. (1979). Intraclass correlations: Uses in assessing rater reliability. *Psychol Bull, 86*, 420-428. doi:10.1037/0033-2909.86.2.420
